# Anti-TRBC1 Antibody-Based Flow Cytometric Detection of T-Cell Clonality: Standardization of Sample Preparation and Diagnostic Implementation

**DOI:** 10.3390/cancers13174379

**Published:** 2021-08-30

**Authors:** Noemí Muñoz-García, Margarida Lima, Neus Villamor, F. Javier Morán-Plata, Susana Barrena, Sheila Mateos, Carolina Caldas, Ana Balanzategui, Miguel Alcoceba, Alejandro Domínguez, Fabio Gómez, Anton W. Langerak, Jacques J. M. van Dongen, Alberto Orfao, Julia Almeida

**Affiliations:** 1 Translational and Clinical Research Program, Centro de Investigación del Cáncer and IBMCC (CSIC-University of Salamanca), Cytometry Service, NUCLEUS, Department of Medicine, University of Salamanca (USAL) and Institute of Biomedical Research of Salamanca (IBSAL), 37007 Salamanca, Spain; noemimg@usal.es (N.M.-G.); fjmoranp@usal.es (F.J.M.-P.); subadelfa@usal.es (S.B.); sheilamateos@usal.es (S.M.); carolina.caldas@usal.es (C.C.); orfao@usal.es (A.O.); 2 Biomedical Research Networking Centre Consortium of Oncology (CIBERONC), Instituto de Salud Carlos III, 28029 Madrid, Spain; villamor@clinic.cat (N.V.); abal@usal.es (A.B.); alcocebasanchez@saludcastillayleon.es (M.A.); 3 Department of Hematology, Laboratory of Cytometry, Hospital de Santo António, Centro Hospitalar do Porto, 4099-001 Porto, Portugal; margaridalima@chporto.min-saude.pt; 4 Unit for Multidisciplinary Research in Biomedicine (UMIB), Abel Salazar Institute of Biomedical Sciences (ICBAS), University of Porto, 4050-313 Porto, Portugal; 5 Department of Pathology, Hematopathology Unit, Hospital Clínic, IDIBAPS, 08036 Barcelona, Spain; 6 Hematology Service, University Hospital of Salamanca, Translational and Clinical Research Program, Centro de Investigación del Cáncer/IBMCC and IBSAL, 37007 Salamanca, Spain; 7 Centro de Salud Miguel Armijo, Sanidad de Castilla y León (SACYL), 37007 Salamanca, Spain; aldominguezb@saludcastillayleon.es (A.D.); fgomezgar@saludcastillayleon.es (F.G.); 8 Department of Immunology, Laboratory Medical immunology, Erasmus MC, University Medical Center Rotterdam, 3015 GD Rotterdam, The Netherlands; a.langerak@erasmusmc.nl; 9 Department of Immunology, Leiden University Medical Center (LUMC), 2333 ZA Leiden, The Netherlands; J.J.M.van_Dongen@lumc.nl

**Keywords:** TRBC1, JOVI-1, T-CLPD, Tαβ-cells, TRBJ1 and TRBJ2, TCRVβ, MRD1

## Abstract

**Simple Summary:**

The anti-TRBC1 antibody JOVI-1 has recently been identified as a flow cytometry marker potentially useful for assessment of T-cell clonality. The aim of this study was to optimize a flow cytometric method for routine use of anti-TRBC1 to assess T-cell clonality and validate it in a large series of normal and pathological samples. Our results showed that the best resolution to accurately identify TRBC1^+^ cells was achieved by adding the CD3 antibody either simultaneously or after TRBC1. In addition, TRBC1^+^/TRBC1^−^ ratios within different Tαβ-cell subsets are provided as expected reference ranges for polyclonal T-cells. Based on the optimized approach here proposed, we detected monoclonal Tαβ-cell populations with high specificity (96%) and a high analytical sensitivity/level of detection (≤10^−4^), when clonal T-cells exhibited immunophenotypic aberrancies. These findings further support and extend previous observations about the utility of TRBC1 for the diagnostic screening and monitoring of clonal Tαβ-cell populations.

**Abstract:**

A single antibody (anti-TRBC1; JOVI-1 antibody clone) against one of the two mutually exclusive T-cell receptor β-chain constant domains was identified as a potentially useful flow-cytometry (FCM) marker to assess Tαβ-cell clonality. We optimized the TRBC1-FCM approach for detecting clonal Tαβ-cells and validated the method in 211 normal, reactive and pathological samples. TRBC1 labeling significantly improved in the presence of CD3. Purified TRBC1^+^ and TRBC1^−^ monoclonal and polyclonal Tαβ-cells rearranged TRBJ1 in 44/47 (94%) and TRBJ1+TRBJ2 in 48 of 48 (100%) populations, respectively, which confirmed the high specificity of this assay. Additionally, TRBC1^+^/TRBC1^−^ ratios within different Tαβ-cell subsets are provided as reference for polyclonal cells, among which a bimodal pattern of TRBC1-expression profile was found for all TCRVβ families, whereas highly-variable TRBC1^+^/TRBC1^−^ ratios were observed in more mature vs. naïve Tαβ-cell subsets (vs. total T-cells). In 112/117 (96%) samples containing clonal Tαβ-cells in which the approach was validated, monotypic expression of TRBC1 was confirmed. Dilutional experiments showed a level of detection for detecting clonal Tαβ-cells of ≤10^−4^ in seven out of eight pathological samples. These results support implementation of the optimized TRBC1-FCM approach as a fast, specific and accurate method for assessing T-cell clonality in diagnostic-FCM panels, and for minimal (residual) disease detection in mature Tαβ^+^ leukemia/lymphoma patients.

## 1. Introduction

T-cell chronic lymphoproliferative disorders (T-CLPD) are uncommon lymphoid malignancies (approximately 10–15% of all peripheral/mature lymphoid neoplasms worldwide) derived from post-thymic T-cells [1,2], which comprise a heterogeneous group of entities with variable clinical behavior [1,3] and biologic features [4,5,6,7,8]. Diagnosis of T-CLPD in cases with lymphocytosis or suspected T-cell populations is often challenging due to the lack of fast and reproducible routine diagnostic assays for T-cell clonality together with the morphologic and immunophenotypic similarities between malignant/clonal T-cells and normal (reactive) polyclonal T-cells in a significant fraction of the patients. This contrasts with assessment of B-cell clonality for which fast flow cytometry (FCM) approaches, through demonstration of (either kappa or lambda) restricted expression of light chain immunoglobulins, have been available for several decades [9]. Therefore, the availability of a similarly simple, fast, and reliable approach for assessment of T-cell clonality would be strongly welcomed.

Currently, FCM-based T-cell receptor Vβ (TCRVβ) repertoire and/or polymerase chain reaction (PCR)-based TRB and/or TRG gene rearrangement analysis assays are used to assess the clonal nature of suspicious T-cell populations in the diagnostic work-up of T-CLPD [10]. However, both approaches show limitations for routine implementation. The TCRVβ-FCM assay is relatively expensive, labor-intensive, provides results which might be difficult to interpret for nonreference centers and unexperienced flow cytometrists (particularly in case of oligoclonal expansions and clones with dim TCR expression), and it has a limited sensitivity [11,12,13]. In turn, TR gene rearrangement analysis by PCR is relatively complex and time-consuming (requires experienced personnel and results are generally not available on the same day), does not provide accurate quantitation of the size of the T-cell clone, and/or lacks simultaneous information about the phenotypic characteristics of the expanded clone, which needs to be discriminated from the background of polyclonal T-cells [14,15]; sometimes it might even require prior enrichment/isolation of the suspicious clonal T-cell population to reach enough sensitivity [14,16,17]. Furthermore, both FCM and PCR assays are not routinely available in many diagnostic laboratories due to the low prevalence of T-CLPD.

Recently, a single antibody (TRBC1-binding monoclonal antibody, clone JOVI−1) against one of the two mutually exclusive TCR β chain constant domains (TRBC1 and TRBC2) randomly selected during rearrangement of the TRB gene, has been proposed as a potential marker for rapid assessment of Tαβ-cell clonality by FCM [18]. Normal, as well as virus-specific Tαβ-cells, show an admixture of TRBC1-positive (37–51% and 36–52% of normal CD4^+^ and CD8^+^ T-cells, respectively) [18,19,20,21,22] and TRBC1-negative (presumably TRBC2 positive) T-cells (polyclonal profile in GeneScan studies), whereas monoclonal Tαβ-cells typically showed restricted (monotypic) TRBC1 expression [18,19,20,21,23,24,25]. Recent reports have further shown the potential utility of this antibody reagent for routine assessment of Tαβ-cell clonality in T-CLPD vs. normal/reactive conditions [18,19,20,21,23,24,25,26]. Despite this, optimal standardization of the technique for routine use in diagnostic laboratories, and interpretation of the results based on normal reference TRBC1^+^/TRBC1^−^ ratios and ranges for both normal and reactive Tαβ-cells (and their subsets), have not been provided. Similarly, the demonstration of both the specificity and (analytical) sensitivity of FCM assessment of the TRBC1-expression profile of Tαβ-cells for detecting clonal Tαβ-cells present at low numbers, including the validation of the TRBC1 assay against the gold standard (i.e., PCR) in normal/reactive vs. pathological samples, are still missing.

In this study, we optimized the TRBC1-based FCM approach for identification of clonal Tαβ-cells by: (i) standardizing the TRBC1 staining protocol; (ii) defining the TRBC1-expression profile (i.e., TRBC1^+^/TRBC1^−^ ratios) of normal (total) Tαβ-cells and their Tαβ-cell subsets, defined according to CD4 and CD8 expression, TCRVβ family expression and T-cell maturation stages, and (iii) evaluating its (analytical) sensitivity and specificity for detection of clonal Tαβ-cells present at minimal disease levels. Our ultimate goal was to standardize and validate the utility of the optimized TRBC1-FCM assay for routine detection of T-cell clonality by FCM in a large cohort of normal and reactive vs. pathological samples.

## 2. Materials and Methods

### 2.1. Patients, Controls and Samples

A total of 211 EDTA-anticoagulated samples (from 211 different subjects) were collected between November 2019 and March 2021, consisting of 192 peripheral blood (PB), nine skin (SK), five bone marrow (BM), four lymph node (LN) and one abdominal mass (AM) specimens. From them, 92 PB samples were collected from adult healthy donors (HD), 10 of whom (11%) showed a clonal Tαβ-cell population, identified for the first time in this study by the new TRBC1 assay, and 32 from subjects with reactive expansions of T lymphocytes. Their mean ages (±1SD) were 43 ± 10 years (y), 52 ± 13 y and 52 ± 21 y, for HD, otherwise-HD with a Tαβ-cell clone (HDc) and individuals with reactive lymphocytosis, respectively. The remaining 87 specimens were obtained from patients with different diagnostic subtypes of T-CLPD (mean age of 64 ± 16 y) (Table 1). 

The precise distribution of samples and the study groups corresponding to the different sets of experiments performed are detailed in Supplementary Material (Supplementary Methods and Figure S1).

### 2.2. General Immunophenotypic Approach

All samples were immunophenotyped using a direct immunofluorescence stain-and-then-lyse technique, based on the EuroFlow standard operating procedures (SOP) for staining of cell surface markers only [27,28,29], with modifications described below for the different conditions tested during optimization of the TRBC1 assay. Immediately after completion of sample preparation, stained cells were measured in FACSCanto II or LSRFortessa X-20 flow cytometers (Becton/Dickinson Biosciences (BD), San Jose, CA, USA) equipped with the FACSDiva^TM^ software (BD), or in a 5-laser Cytek^®^ Aurora spectral flow cytometer (Cytek Biosciences, Fremont, CA, USA), using the SpectroFlo^®^ software (Cytek Biosciences, Fremont, CA, USA). Instrument setup, calibration and daily quality control, as well as monitoring, were performed according to well-established EuroFlow protocols [28,29]. For data analysis, the INFINICYT^TM^ software (Cytognos, Salamanca, Spain) was used.

### 2.3. Optimization of TRBC1 Staining for Flow Cytometry

In a first step, competition assays with distinct purified CD3 clones (SK7 or UCHT1) and fluorochrome-conjugated CD3 clones (SK7, REA613 and UCHT1) were performed in paired aliquots of six PB samples from HDs (Table S1A), as detailed in Supplementary Methods. 

The potential steric hinderance between surface membrane CD3 and TRBC1 was subsequently tested in paired aliquots of 11 HD PB samples under four different staining conditions: (a) staining with TRBC1 only; and with both CD3 and anti-TRBC1 reagents where CD3 was added; (b) 10 min after the anti-TRBC1 reagent; (c) simultaneously or (d) 10 min before TRBC1. Different fluorochrome-conjugated anti-TRBC1 and CD3 reagents were tested. Additional conditions were evaluated to compare the number of washing steps (1 vs. 2) and the time of staining after sample collection (immediately and at 24 h, 48 h and 72 h), as detailed in Supplementary Material (Supplementary Methods and Table S1).

The steric interaction between TCRVβ and TRBC1 was also evaluated in two PB samples from HDs for each of the 24 TCRVβ families included in the IOTest^®^ Beta Mark TCRVβ Repertoire Kit (Beckman Coulter, Brea, CA, USA) under three different incubation conditions (Supplementary Methods and Table S1E). 

The sources and specificities of all Mab reagents used for the immunophenotypic assays are detailed in Table S2. For evaluation of TRBC1 staining, both the percentage of TRBC1-positive cells from all TCRαβ-cells, as well as their stain index (SI), were calculated [30]. The gating strategy employed for the identification of the different TRBC1-positive T-cell populations was carried out by selecting the most intense data peak in either a single TRBC1 parameter histogram or a 2 D (CD3 or CD4 or CD8 vs. TRBC1) dot-plot.

### 2.4. PCR-Based Detection of TRBJ1 or TRBJ2 Gene Rearrangements in FACS-Sorted Tαβ-Cell Populations

**Figure 1:**
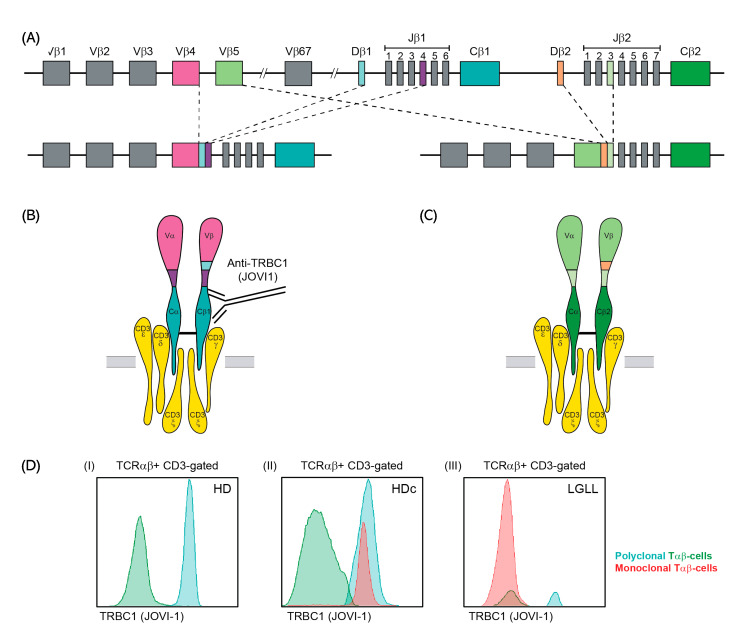
Schematic representation of TRB gene rearrangement and interpretation of the TRBC1 antibody (JOVI-1 clone)-based flow cytometry approach: (**A**) Mutually exclusive TRBC selection during TRB gene rearrangement in the thymus; (**B**,**C**) Representation of the two resulting TRB complex structures, composed of either the TRBC1 (**B**) or the TRBC2 (**C**) proteins, and specific binding of the anti-TRBC1 antibody to TRBC1 but not to TRBC2; (**D**) Illustrative histograms of TRBC1 staining of blood Tαβ-cells from: (I) one representative adult HD, showing the bimodal TRBC1 expression pattern, typical of polyclonal Tαβ-cells (TRBC1^+^ in light blue and TRBC1^−^ in green); (II) one HDc showing a minor TRBC1^+^ (clonal) Tαβ-cell population (in red), among a majority of polyclonal Tαβ-cells; and (III) one LGLL case with a major population of TRBC1^−^ monoclonal Tαβ-cells (in red) with a minor background of polyclonal Tαβ-cells. Monoclonal T-cells were selected by the presence of a phenotypic aberrancy and/or expression of a single TCRVβ family, both in HDc (e.g., CD8^+^TCRVβ16^+^) and in LGLL (e.g., CD8^+^CD279^++^) cases. Abbreviations (alphabetical order): HD, healthy donor; HDc, healthy donor with a small Tαβ-cell clone in blood; LGLL, large granular lymphocytic leukemia.

Confirmation of mutually exclusive TRBC1 vs. TRBC2 gene usage in different populations of Tαβ-cells with a TRBC1-positive vs. TRBC1-negative phenotype was assessed in genomic DNA extracted from 95 FACS-sorted (FACSAria III, BD Biosciences, San Jose, CA, USA) CD3^+^ TαβCD4^+^ and TαβCD8^+^ cell populations from 28 different individuals (purity ≥ 95%) that showed optimal PCR amplification of the TRBJ gene product based on the presence of single TRBJ1 vs. double TRBJ1+TRBJ2 gene rearrangement patterns, respectively. For this purpose, well-established EuroClonality/BIOMED-2 primers, protocols and criteria [15] were used, based on the fact that both TRBC genes are preceded by the corresponding TRBJ genes (TRBC1 is preceded by six TRBJ1 genes while TRBC2 is preceded by seven TRBJ2 genes; Figure 1) [31,32]. For these studies, either the GenElute^TM^ Mammalian Genomic DNA Miniprep Kit (Sigma-Aldrich, St Louis, MO, USA) or the Genomic Tissue DNA Kit (ThermoFisher Scientific, Waltham, MA, USA) were used, as per the instructions of the manufacturers.

### 2.5. Analysis of the TRBC1^+^/TRBC1^−^ Ratio in Distinct Subsets of Normal Tαβ-Cells Defined by the TCRVβ Family Expressed and Their Maturation Stage

Both within total Tαβ-cells and their major (TαβCD4^+^, TαβCD8^+^, TαβDP and TαβDN cells) subsets, the TRBC1^+^/TRBC1^−^ ratio within each cell population defined by the expression of the different TCRVβ families was analyzed in a group of 27 PB samples (12 HD, 10 patients with reactive lymphocytosis and five HDc whose clonal T-cell populations were excluded from the analysis), stained with the IOTest^®^ Beta Mark TCRVβ Repertoire Kit (Beckman Coulter), following the CD3-10 min-TRBC1 and the TRBC1-10 min-TCRVβ conditions described in Supplementary Material (Table S1F).

In turn, the TRBC1^+^/TRBC1^−^ ratio for the distinct maturation-associated compartments of normal Tαβ-cells (i.e., naïve, central memory, transitional memory, effector memory, early effector and terminal effector T-cells and regulatory T-cells), identified according to the phenotypic profile shown in Table S3, was investigated in PB samples from 10 HD (Table S1G).

### 2.6. Assessment of T-Cell Clonality on FACS-Sorted Cell Populations for Patients with T-CLPD vs. Reactive Lymphocytosis and Healthy Donors

The (mono)clonal vs. polyclonal nature of Tαβ-cell populations from patients with T-CLPD and reactive lymphocytosis, as well as HD (including the small Tαβ-cells clones from HDc, and the different maturation-associated compartments of Tαβ-cell populations from HD) was assessed in highly-purified (≥95% purity) FACS-sorted cells (purified from 3–5 mL of whole blood using a FACSAria-III cell sorter, BD Biosciences, San Jose, CA, USA), as previously described [10,14,15], based on the presence (vs. absence) of single vs. a few dominant TRB and/or TRG gene rearrangements for clonal and oligoclonal/polyclonal cell subsets, respectively. In one T-cell large granular lymphocyte leukemia (T-LGLL) patient and five T-cell prolymphocytic leukemia (T-PLL) patients, T-cell clonality was further established on purified cells through confirmation of the presence of *STAT3* (somatic) mutations and the demonstration of *TCL1* (or *MTCP1*) gene translocations, respectively [1,33,34]. 

### 2.7. Validation of the TRBC1-FCM Assay against Conventional Molecular and FCM Techniques for Detection of Clonal Tαβ-Cells

The specificity of the TRBC1-FCM assay for identification of (true) polyclonal vs. monoclonal cell populations was validated using different fluorochrome conjugates, BV421, BV480, dyomics (Dy) 634 or FITC, of the anti-TRBC1 antibody reagent in 24 poly/oligoclonal and 93 monoclonal samples, as determined by the TCRVβ Repertoire FCM Kit, PCR [15] and/or by the presence of specific gene mutations (e.g., *STAT3* mutations in T-LGLL). Of the 89/93 (mono)clonal samples (96%) a final WHO diagnosis [1] was available in 79 T-CLPD cases, while the other 10 cases concerned HDc samples.

### 2.8. Serial Dilution Experiments of Pathological Tαβ-Cells in Normal Blood Cells

The sensitivity of the TRBC1-FCM approach for detecting clonal Tαβ-cells was determined using both real and in silico serial dilutional experiments of PB samples (or FCM events) from T-CLPD tumor cells in normal PB. For this purpose, a total of eight experiments were performed in six patients, including parallel real and in silico dilutions in two out of six cases, and four in silico dilutions carried out in the other four cases. In all cases, dilution of the T-CLPD patient blood in PB samples from HD at 1:10, 1:100, 1:1000 and 1:10,000 tumor cells/normal cell ratios were performed. In each case, the pathological population was identified based on the presence of an aberrant phenotype (e.g., CD5^−^) plus restricted expression of a single TCRVβ family, without using the TRBC1 staining for selection of the suspicious cell population. Per dilutional experiment ≥500,000 target cells were acquired and a minimum of 50 clustered cellular events were required to consider them as a cell population.

### 2.9. Statistical Methods

The nonparametric Mann–Whitney U test and the Spearman’s correlation test (for continuous variables), together with the Fisher exact test (for categorical variables), were used for group comparisons, performed with the IBM-SPSS software (v25.0; IBM, Armonk, NY, USA) and/or GraphPad Prism software (v5.01; GraphPad, San Diego, CA, USA). Prior to the comparisons, conventional normality tests (Q-Q plots, P-P plots and Kolmogorov-Smirnov test) were used to check for the normal (Gaussian) distribution of individual variables. *p*-values ≤ 0.05 were considered to be associated with statistical significance.

## 3. Results

### 3.1. Optimization of TRBC1 Staining by FCM

**Figure 2:**
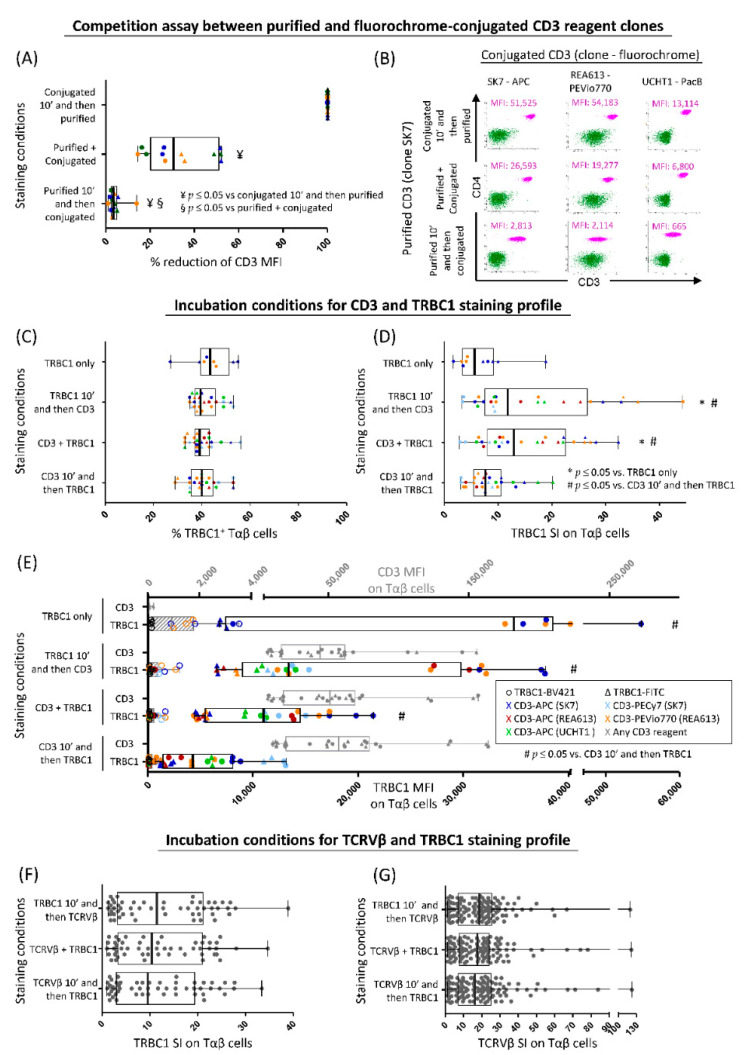
Impact of CD3 and TCRVβ antibody staining on the expression profile of TRBC1. (**A**) Competition assays performed in 6 paired PB samples stained with different fluorochrome-conjugated CD3 clones (*n* = 3, SK7, REA613 and UCHT1) and unconjugated (=purified) CD3 reagents (SK7 and UCHT1) clones, tested under three different incubation conditions: (a) addition of the conjugated reagent first followed by a 10 min incubation before the unconjugated antibody was added; (b) simultaneous addition of the fluorochrome-conjugated and unconjugated antibody reagents; and (c) addition of the unconjugated reagent first (10 min before) followed by the fluorochrome-conjugated reagent (*p*-value ≤ 0.05 for ^¥^ purified + conjugated vs. conjugated 10′ and then purified reagent, and for ^§^ purified 10′ and then conjugated vs. purified + conjugated reagent). (**B**) Illustrative graphical dot-plots of TαβCD4^+^ cells (pink dots) and B cells (dark green dots) stained in competition assays described in A; (**C**–**E**) percentage of TRBC1^+^ Tαβ-cells and the SI and MFI of TRBC1^+^ Tαβ-cells stained under four different conditions (staining with TRBC1 only vs. both CD3 and TRBC1 where CD3 was added 10′ after, simultaneously or 10′ before TRCB1) (*p*-value ≤ 0.05 for * any condition vs. TRBC1 only and for ^#^ any condition vs. CD3 10′ and then TRBC1); (**F**–**G**) Comparison of 3 different incubation conditions for the TRBC1 and TCRVβ antibody reagents are displayed as SI for TRBC1 and TCRVβ reagents as readout, respectively. Symbols and color codes in panels A-E are as follows: TRBC1-FITC, triangle; TRBC1-BV421, circle; CD3-APC-SK7, dark blue; CD3-PECy7-SK7, light blue; CD3-APC-REA613, dark red; CD3-PEVio770-REA613, orange; and CD3-APC-UCHT1, light green; gray identifies any CD3 reagent. In the box-plot graphics, dots correspond to results from individual experiments while notched boxes represent 25th and 75th percentile values, lines inside the box correspond to median values (50th percentile) and whiskers represent minimum and maximum values. Stain index (SI) was calculated as (MFI_PP_ − MFI_NP_)/2 × rSD_NP_ where MFI represents median fluorescence intensity values (arbitrary units scaled from 0 to 262,144), rSD is the robust standard deviation, and PP and NP are used as abbreviations for the TRBC1 positive and the TRBC1 negative Tαβ-cell populations, respectively. Other abbreviations (alphabetical order): APC, allophycocyanin; BV, brilliant violet; Cy, cyanin; FITC, fluorescein-5-isothiocyanate; PacB, pacific blue; PB, peripheral blood; PE, phycoerythrin.

A significant decline of median fluorescence intensity (MFI) values for the fluorochrome-conjugated SK7, REA613 and UCHT1 CD3 clones was observed when the purified (unconjugated) SK7 and UCHT1 clones had been added prior to staining for all antibody clone combinations tested in the competition assays, compared to both simultaneous addition of a mixture of the purified (unconjugated) and fluorochrome-conjugated antibodies and particularly, addition of fluorochrome-conjugated CD3 reagents 10 min before the purified antibodies (Figure 2A,B). These results support that all clones tested recognize the same (or overlapping) CD3 epitope, as previously reported for e.g., SK7 and UCHT1 [35].

Staining with TRBC1 in the absence of CD3 was associated with clear staining of a fraction of the lymphocytes, but unwanted levels of background fluorescence in another subset of the lymphoid T-cells. This led to relatively low SI (Figure 2C, D). In turn, staining for TRBC1 in the presence (vs. absence) of CD3 was associated with a significantly improved discrimination between TRBC1^+^ vs. TRBC1^−^ Tαβ-cells and significantly higher SI; despite this, absence of CD3 did not impact the percentage of TRBC1^+^ Tαβ-cells identified (Figure 2C,D). When we compared different incubation conditions for the CD3 and TRBC1 double-staining on the TRBC1 expression profile in terms of both SI and MFI of the TRBC1^+^ and TRBC1^−^ Tαβ-cell populations (Figure 2D,E), we found that addition of TRBC1 either prior or simultaneously to (but not after) CD3 was associated with the highest TRBC1 SI on Tαβ-cells and, thereby, a more clear discrimination between TRBC1^+^ and TRBC1^−^ Tαβ-cells (Figure 2D) was observed, with progressively decreased MFI values of TRBC1^+^ at the expense of greater (*p* < 0.05) CD3 MFI values in Tαβ-cells (Figure 2E). Of note is that a similar staining profile was observed for the different CD3 clones, as well as CD3 and TRBC1 fluorochrome conjugates tested (Figure 2C–E and Figure S2). 

In contrast to the lower TRBC1 SI found when CD3 was added prior to TRBC1, no impact (*p* > 0.05) was observed on the TRBC1 SI when TCRVβ reagents were added first (prior to TRBC1), for any of the other incubation conditions tested; likewise, the TCRVβ SI was similar for all staining conditions evaluated (Figure 2F,G and Figure S2).

Additional testing concerning the number of washing steps showed that performing 1 vs. 2 washes after adding the lysing solution did not have an impact on the TRBC1 SI (Figure S2). Similarly, TRBC1-FITC labeling did not decrease with time (vs. 0 h) even when sample staining was performed 72 h after blood collection, while the TRBC1-BV421 SI was significantly reduced when samples were stained 48 h after collection or later (Figure S2). 

### 3.2. TRBJ Gene Rearrangements in FACS-Sorted TRBC1^+^ and/or TRBC1^−^ Tαβ^+^-Cell Populations

FACS-sorted TRBC1^+^ populations of total Tαβ^+^ cells showed functionally rearranged TRBJ1 sequences in 44 of 47 cell populations (94%) investigated, while rearrangements confirming the presence of the TRBJ1+TRBJ2 gene rearrangements were found in the remaining three of 47 (6%) cell populations. In contrast, FACS-sorted TRBC1^−^ cell populations showed rearranged genes containing both the TRBJ1 and TRBJ2 sequences in all 48 (100%) cell populations tested at the DNA level, regardless of their clonal status (monoclonal, oligoclonal or polyclonal populations) and, therefore, of the sample origin (HD, reactive lymphocytosis, HDc or T-CLPD) (Table 2).

### 3.3. Ranges for Polyclonal (Normal and Reactive) Tαβ-Cells and Major Tαβ-Cell Populations

Total Tαβ-cells from normal PB (*n* = 65 HD) showed a mean (±SD) percentage of TRBC1^+^ cells of 39 ± 5.8%, which translated into a TRBC1^+^/TRBC1^−^ ratio of 0.63 ± 0.062. Of note is this TRBC1^+^/TRBC1^−^ ratio varied significantly for some of the major Tαβ-cell subsets in the same set of (normal) samples: TαβCD4^+^, 0.72 ± 0.062 (*p* = 0.003); TαβCD8^+^, 0.50 ± 0.081 (*p* < 0.0001); TαβDP, 0.51 ± 0.13 (*p* = 0.001); and TαβDN, 0.38 ± 0.11 (*p* < 0.0001) (Table S4). In parallel, the percentage of TRBC1^+^ cells and the corresponding TRBC1^+^/TRBC1^−^ ratios were also calculated for the same major PB subsets of Tαβ-cells from subjects with reactive lymphocytosis (*n* = 18), with a similar distribution to that observed for normal PB Tαβ-cells (Table S4). Thereby, the percentage of TRBC1^+^ cells and the TRBC1^+^/TRBC1^−^ ratio of HD plus reactive lymphocytosis (*n* = 83) was calculated and used to derive normal range values for polyclonal (normal and reactive) cells (Table 3). For this purpose, the mean ± 3 standard deviations, which define intervals where 99.73% of TRBC1^+^/TRBC1^−^ ratios from polyclonal cells fall, were used as cut-off values for defining monoclonal vs. polyclonal Tαβ-cell profiles (Table 3). Based on these cut-offs, the normal range for the TRBC1^+^/TRBC1^−^ ratio for total Tαβ-cells extended from 0.25 to 1.4; for TαβCD4^+^ cells it ranged from 0.31 to 1.6, and for TαβCD8^+^ from 0.091 to 1.6 (Table 3). 

### 3.4. TRBC1^+^/TRBC1^−^ Ratio of Normal Polyclonal Tαβ-Cells and Their TCRVβ and Maturation-Associated Subsets in Normal Blood

**Figure 3:**
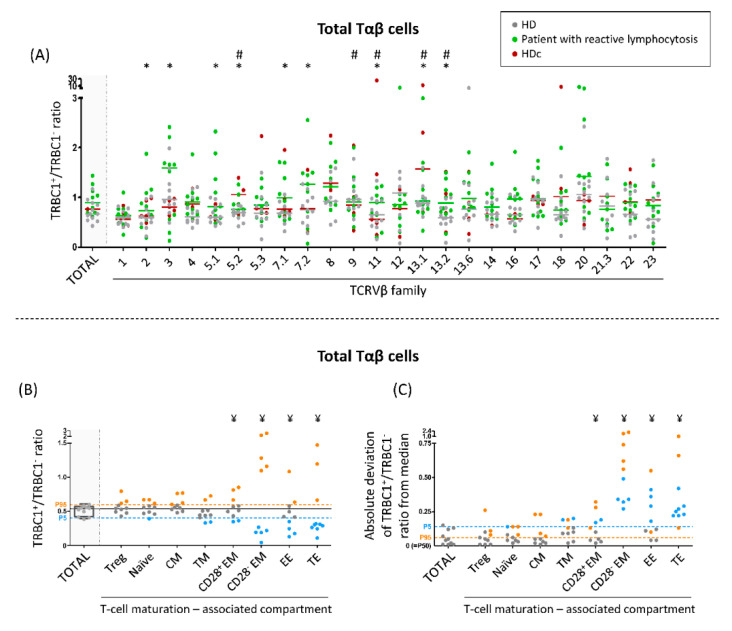
TRBC1^+^/TRBC1^−^ ratio of normal total Tαβ-cells according to the specific TCRVβ family member expressed and their maturation stage. (**A**) TRBC1^+^/TRBC1^−^ ratio of total Tαβ-cells within each of the different TCRVβ families identified by immunophenotype in 12 HD (gray dots), 10 patients with reactive lymphocytosis (green dots) and 5 otherwise healthy individuals showing a clonal expansion of Tab cells in blood (HDc, red plots). In these latter subjects, the TCRVβ clonal population was removed from analysis. Colored horizontal lines are median values of the corresponding group of subjects (*p*-value ≤ 0.05 for * reactive lymphocytosis vs. HD and for ^#^ HDc vs. HD) (**B**,**C**) TRBC1^+^/TRBC1^−^ ratio observed among normal Tαβ-cells from 10 HD, distributed into different maturation-associated compartments, represented both in individual ratio values per maturation stage (**B**) and their deviation from the median value (percentile 50 = 0; (**C**)) (^¥^ *ρ*-value ≤ 0.05 vs. total Tαβ-cells) In all panels, dots correspond to results from individual experiments while notched boxes represent 25th and 75th percentile values, lines inside the box correspond to median values (50th percentile) and whiskers represent minimum and maximum values. The continuous horizontal and dotted lines that cover the entire graph correspond to median values (percentile 50) and both the 5th and 95th percentiles (P5 and P95), respectively. Cases below P5 are depicted in blue, while cases above P95 are colored as orange dots. Abbreviations (alphabetical order): CM, central memory; DN, double negative (TαβCD4^−^CD8^−/lo^) Tαβ-cells; DP, double positive (TαβCD4^+^CD8^+^) Tαβ-cells; EE, early effector; EM, effector memory; HD, healthy donor; HDc, healthy donor with a small Tαβ-cell clone in blood; TE, terminal effector; TM, transitional memory; Treg, regulatory T-cells.

For every TCRVβ subset of Tαβ-cells from HD, a bimodal distribution was observed, with both TRBC1^+^ and TRBC1^−^ cells. However, TRBC1^+^ and TRBC1^−^ Tαβ-cells were differentially distributed according to the TCRVβ family expressed (Figure 3A). Thus, the median TRBC1^+^/TRBC1^−^ ratio observed for Tαβ-cell subsets that expressed one of the 24 TCRVβ families ranged from 0.56 to 1.1 (Figure 3A). As expected, a bimodal TRBC1 expression profile was also observed when the analysis was restricted to the major subsets of Tαβ-lymphocytes expressing different TCRVβ families (Figure S3). Thus, TRBC1^+^/TRBC1^−^ ratios for the major Tαβ-cell subsets of TαβCD4^+^, TαβCD8^+^, TαβDP and TαβDN cells, expressing different TCRVβ families ranged from 0.61 to 1.2, from 0.33 to 0.85, from 0.25 to 1.4 and from 0.25 to 0.77, respectively (Figure S3). 

Subsequently, we investigated the TRBC1^+^/TRBC1^−^ ratio distribution within different maturation-associated compartments of Tαβ-cells from a subgroup of 10 HD (Figure 3B). Our results showed that at earlier maturation stages (i.e., naïve, central memory and transitional memory cells as well as regulatory Tαβ-cells) most samples were within the 5th and 95th percentiles observed for the total population of Tαβ-cells in normal/reactive blood. In contrast, at the more mature stages of effector memory, early effector and terminal effector Tαβ-cells, a statistically significant number of cases were outside the normal (5th and 95th percentile) range observed for total Tαβ-cells (i.e., more dispersed TRBC1^+^/TRBC1^−^ ratios) (Figure 3B,C). A similar profile distribution was found for TRBC1^+^/TRBC1^−^ ratios of the different Tαβ-cell subsets, meaning that in most samples earlier maturation stages (naïve and central/transitional memory stages) were inside the 5th and 95th percentiles observed for the total population of TαβCD4^+^, TαβCD8^+^, TαβDP and TαβDN cells (Figure S3), while more mature cell subsets mostly fell outside the 5th and 95th percentile values of total Tαβ-cells in blood samples from HD (Figure S3).

### 3.5. TRBC1^+^/TRBC1^−^ Ratio of Polyclonal Tαβ-Cells Expressing Different TCRVβ Families in Patients with Reactive Lymphocytosis and HDc Blood

A bimodal distribution of TRBC1^+^ and TRBC1^−^, differentially distributed according to the specific TCRVβ family expressed, was observed among Tαβ-cells and their major subsets in reactive lymphocytosis and HDc samples (Figure 3A and Figure S3). Thus, the median TRBC1^+^/TRBC1^−^ ratio observed for each of the 24 TCRVβ families of Tαβ-cells ranged from 0.64 to 1.6 in blood of patients with reactive lymphocytosis and from 0.54 to 1.6 in HDc (Figure 3A), with nine of 24 values (38%) in reactive lymphocytosis cases and five of 24 values (21%) in HDc cases samples outside the 5th and 95th percentiles as observed in HD (Figure 3 A). TRBC1^+^/TRBC1^−^ ratios for the different Tαβ-cell subsets (TαβCD4^+^, TαβCD8^+^, TαβDP and TαβDN cells) expressing different TCRVβ families from reactive lymphocytosis and HDc are detailed in legend to Figure S3.

### 3.6. Comparison between the TRBC1-FCM Assay and Conventional TCRVβ-FCM and/or Molecular Techniques for Assessment of Tαβ-Cell Clonality

Upon comparing the TRBC1-FCM assay with the reference TCRVβ-FCM and/or molecular techniques for assessment of Tαβ-cell clonality, concordant results were found in 112 of 117 cases (96%) (Table 4). Concordant cases corresponded to 21 of 24 poly/oligoclonal cases (87%) that showed a polytypic TRBC1 profile by FCM and 91 of 93 monoclonal samples (98%) that displayed a monotypic TRBC1-FCM pattern. There were only five of 117 discrepant cases (4.3%), either because cases that were classified as poly/oligoclonal by PCR showed a monotypic pattern of TRBC1 (*n* = 3) or because monoclonal cases by PCR showed a polytypic TRBC1 pattern (*n* = 2). Additional information on these five discrepant cases is provided in detail in Table S5 and Figure S4 and discussed below. Thus, two of three PCR polyclonal samples (cases #1 and #2) in whole blood, showed a monotypic TRBC1 expression with TRBC1^+^/TRBC1^−^ ratios of <0.01 and >99 within the population(s) of phenotypically aberrant (CD2^lo^ CD3^lo^ CD5^−/lo or ++^) Tαβ-cells that represented 2.5% and 5.9% of all blood leukocytes, respectively. In the remining (discordant) sample (case #3) classified as oligoclonal by PCR (analyzed on FACS-purified cells), a monotypic expression of TRBC1 with a TRBC1^+^/TRBC1^−^ ratio of 8.0 was observed. This latter cell population phenotypically consisted of terminal-effector cytotoxic Tαβ-cells in the absence of an immunophenotypically aberrant phenotype. Conversely, two samples (cases #4 and #5) analyzed in whole PB were considered monoclonal by PCR but showed a polytypic pattern of expression of TRBC1 by FCM with TRBC1^+^/TRBC1^−^ ratios of 0.45 and 0.37, respectively. One of these was diagnosed as reactive T-cell lymphocytosis associated with acute Epstein-Barr virus (EBV) infection (case #4) while the second was unclassifiable (case #5) in the absence of definitive diagnostic criteria for T-LGLL (Table S5 and Figure S4).

Patients that could be finally classified into a precise WHO diagnostic category of T-CLPD (*n* = 79), as well as HD in whom a minor population of clonal Tαβ-cells was detected in blood in this study (*n* = 10), were divided into two groups according to their TRBC1 expression profile for the clonal Tαβ-cells: TRBC1^+^ vs. TRBC1^−^ (Table 5). Interestingly, while in T-PLL (postulated to derive from naïve/central memory cells) TRBC1^+^ only represented three of 10 (30%) cases investigated, among primary cutaneous T-cell lymphoma (PCTCL)-Sézary syndrome (SS) TRBC1^+^ cases represented 80% (12 of 15) of the patients, respectively (Table 5). In turn, a frequency of TRBC1^+^ cases of 50% and 56% was found among PCTCL-mycosis fungoides (MF) (three of six patients) and T-LGLL cases (22 of 39 patients) (Table 5). The TRBC1 expression pattern in other diagnostic categories of T-CLPD is anecdotal, due to the low number of cases investigated. Among HDc, TRBC1 was expressed in nine of 10 (90%) cases identified (Table 5). 

### 3.7. Utility of TRBC1 for Sensitive FCM Detection of Clonal Tαβ-Cellsi in Serial Dilution Experiments of Pathological Tαβ-Cells in Normal Blood Cells 

**Figure 4:**
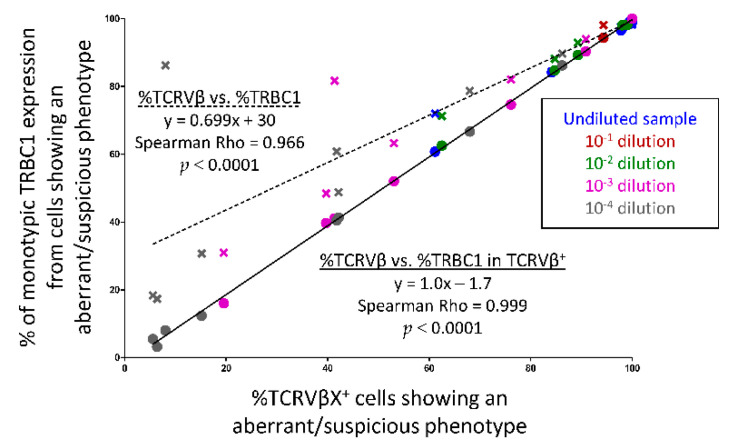
Sensitivity of the TRBC1 marker for detection of clonal Tαβ-cells in serial dilutional experiments of clonal tumor cells in normal blood cells. Scatter plot showing the correlation between the percentage of TCRVβ^+^ vs. TRBC1^+^ cells (crosses and dotted line) and between the percentage of TCRVβ^+^ vs. (TRBC1^+^ and TCRVβ^+^) clonal cells (circles and solid line) within a specific T-cell population showing an aberrant/suspicious phenotype for both undiluted (total) samples and serially diluted (from 10^−1^ to 10^−4^) samples. TCRVβX refers to any of the specific (clonal) TCRVβ family expressed by each case (e.g., TCRVβ22). For each correlation, the estimated linear regression equation, the Spearman’s Rho correlation coefficient and the corresponding *p*-values are shown (based on 8 dilution experiments).

Serial dilution experiments (*n* = 8) of PB clonal Tαβ-cells in normal leukocytes, performed both directly and in silico (Figure S5), showed a high degree of correlation between the percentage of clonal Tαβ-cells identified among cells that displayed an aberrant/suspicious phenotype by monotypic expression of TRBC1 vs. expression of a specific TCRVβ region (R^2^ = 0.966; *p* < 0.001) with a sensitivity of at least 10^−4^ in seven of eight (88%) dilutional experiments (Figure 4). Further identification of clonal Tαβ-cells based on both the pattern of expression of TRBC1 and a specific TCRVβ family (vs. TRBC1 alone) showed a slightly improved correlation (R^2^ = 0.999; *p* < 0.0001), with a sensitivity of at least 10^−4^ in eight of eight (100%) experiments (Figure 4).

## 4. Discussion

Several recent reports have proposed the introduction of the TRBC1-based FCM assay as a potentially useful approach to assess Tαβ-cell clonality in the diagnostic work-up of patients suspicious of T-CLPD [18,19,20,21,23,24,25,26]. Despite this, optimization of the antibody staining conditions, as well as reference ranges for normal and reactive polyclonal Tαβ-cells and their major subsets, together with the sensitivity and specificity of the TRBC1 assay for detecting clonal Tαβ-cells, remain to be fully established prior to its diagnostic routine use. Here we defined the most appropriate staining conditions to obtain the best resolution between TRBC1^+^ and TRBC1^−^ Tαβ-cells. In addition, we established, for the first time, reference TRBC1^+^/TRBC1^−^ ranges in PB for normal and reactive polyclonal Tαβ-cells and their major Tαβ-cell populations. At the same time, we provide preliminary data on the distribution of TRBC1^+^ and TRBC1^−^ Tαβ-cells according to the specific TCRVβ family expressed by total Tαβ-cells (and their major subsets) and their maturation stage. Finally, we confirm and extend on previous observations [18,21,25,26] about the analytical sensitivity and specificity of the assay for detecting monoclonal vs. poly/oligoclonal Tαβ-cell populations, even when present at low frequencies in blood.

Since both CD3 and anti-TRBC1 antibodies recognize physically close epitopes of the CD3/TCRαβ complex [36,37], we first tested the potential steric interaction between both groups of antibody reagents for optimization of the TRBC1 staining. Our results showed that addition of CD3 blocked the (low affinity/unspecific) binding of the anti-TRBC1 reagent to TRBC1^−^ (i.e., TRBC2^+^) Tαβ-cells [38], while similar percentages of TRBC1^+^ cells were observed in the absence vs. presence of the CD3 Mab. This translated into an improved resolution between TRBC1^+^ and TRBC1^−^ cells when CD3 was added either simultaneously or after (but never before) the anti-TRBC1 antibody. These results were consistent across the different CD3 clones tested (i.e., SK7 and UCHT1) and CD3 fluorochrome-conjugated reagents. It should be noted that in some PB samples from HD, a population of Tαβ cells apparently showing very dim expression of TRBC1 was detected, even at different centers, (e.g., Salamanca and Porto), without paralleled low expression of CD3 (data not shown); this could be due to nonspecific labeling or any other nonidentified technical issue related to CD3/TCRVβ and TRBC1 interactions, this population being here considered as TRBC1^−^. Further studies in FACS-sorted populations are needed to confirm that these cells are indeed TRBC1^−^ (i.e., TRBC2^+^). Of note is that while the TRBC1 staining profile remained stable for up to 48 hours when anti-TRBC1-FITC was used, decreased TRBC1 labelling was observed for samples aged >48 h before staining with the TRBC1-BV421 antibody. These results are in line with previous observations pointing out the need to stain fresh (<48 h) samples, also for the CD3-TRBC1 antibody pairs [27,39].

Once staining had been optimized, we subsequently validated the specificity of the TRBC1-based FCM approach based on the demonstration of TRBJ1 gene rearrangements in highly-purified TRBC1^+^ cell populations (either from monoclonal and oligo/polyclonal cases) vs. a double TRBJ1+TRBJ2 gene rearrangement pattern in purified TRBC1^−^ Tαβ-cells, regardless of their clonal nature. Overall, our results showed a high degree of correlation between the pattern of TRBC1 protein expression and the TRBJ1 vs. TRBJ1+TRBJ2 gene rearrangement profile, except for a few discrepant samples. Of note is that such discrepant samples systematically consisted of TRBC1^+^ Tαβ-cells by FCM that displayed double TRBJ1+TRBJ2 gene rearrangements by PCR, which might be due to the presence of incomplete TRB gene rearrangements in the first allele (TRB J1-C1 and TRB J2-C2), together with a complete rearrangement of TRBJ1 on the second allele [15,40,41,42], as demonstrated here. In turn, all TRBC1^−^ populations by FCM showed a first nonproductive or incomplete TRBJ1 rearrangement followed by a productive rearrangement of TRBJ2 at the DNA level of the same chromosome allele [15,40,41,42], which was fully consistent with the FCM assay results.

Similarly to the kappa/lambda ratio in B-CLPD [43,44], the availability of reference TRBC1^+^/TRBC1^−^ ratio ranges for polyclonal Tαβ-cells, including both normal and reactive cells, is critical for routine implementation of the new TRBC1-based FCM assay in the diagnostic work-up of T-CLPD. Thus, several studies have previously reported percentage values of TRBC1^+^ cells within total Tαβ-cells and their major TCD4^+^ and TCD8^+^ populations in normal blood samples [18,20,21]. However, different ranges are reported in these studies, due to the different nature of control samples used (HD vs. reactive blood), the limited numbers of samples investigated and/or the use of different confidence intervals (e.g., 95% CI vs. 99.7% CI) to define cut-offs for T-cell clonality. Here we defined reference TRBC1^+^/TRBC1^−^ ranges for polyclonal Tαβ-cells and all major Tαβ-cell populations, based on the largest cohort of controls reported so far (including both HD and reactive lymphocytosis patients). In addition, more strict cut-off values with larger confidence intervals for normal cells were used for identification of clonal Tαβ-cell expansions. Of note is that with the optimized TRBC1-FCM approach used here, small Tαβ-cell clones were detected in a significant fraction of all HD investigated, in line with previous findings by Horna et al. [23,24,25]. Further molecular analysis performed here on purified suspicious Tαβ-cells populations from these otherwise healthy individuals confirmed that they systematically corresponded to clonal cells by PCR. The significance of the presence of these T-cell clones in blood remains to be elucidated, but they could be either the result of physiologically normal immune activation of T-cells [24,26], similar to the monotypic expression of kappa or lambda in B-cells that can also be seen in skewed immune responses, without additional abnormal marker expression [44], or persistent clonal (immunosenescent) cells that does not necessarily imply malignancy, in a similar way to low-count monoclonal B lymphocytosis [45].

In order to determine whether the above reference ranges could also be applied to other major and minor Tαβ-cell subsets, we further investigated for the first time the distribution of TRBC1^+^ cells within the different TCRVβ populations and maturation-associated subsets of Tαβ-cells. Although different median TRBC1^+^/TRBC1^−^ ratios were found among cells expressing each of the TCRVβ families identified (especially in reactive cases), TRBC1 expression appeared to be independent of the specific TCRVβ family expressed among HD, as well as HDc and reactive lymphocytosis patients, i.e., a bimodal pattern was found for all families. These findings support the use of the TRBC1 expression profile as a surrogate marker of clonality in Tαβ-cells and their major subsets, independently of the specific TCRVβ family expressed. In contrast, here we report for the first time significantly skewed TRBC1^+^/TRBC1^−^ ratios depending on the maturation stage of Tαβ-cells. This is in contrast with previous findings by other groups who did not find differences in the percentage of TRBC1^+^ cells among distinct maturation-associated subpopulations of Tαβ-cells, as defined by the pattern of expression of CD5, CD7 and/or CD26 [20,21,24]. Thus, the overall normal TRBC1^+^/TRBC1^−^ ratio ranges of naïve, central memory, transitional memory and regulatory Tαβ-cell subsets overlapped with those of the whole Tαβ-cell populations and/or their major TαβCD4^+^ and TαβCD8^+^ subsets, whereas more mature populations of CD28^+^ and particularly CD28^-^ effector memory, early effector, and terminal effector cells of HD displayed more heterogeneous and clear skewed TRBC1^+^/TRBC1^−^ ratios compared with those of total Tαβ-cells, regardless of the specific subset of, for example, TαβCD4^+^ or TαβCD8^+^ cells. These findings support an increasingly higher degree of oligoclonality associated with a progressively narrower TR repertoire, along the maturation of blood Tαβ-cells, due to the accumulation of effector T-cells specific for a relatively more limited number of antigens, including antigens from viruses that persist in the organism, such as EBV and cytomegalovirus [46]. The fact that the TRBC1^+^/TRBC1^−^ ratio of more mature Tαβ-cell populations usually deviates from those of total Tαβ-cells from the same subject should be considered in the diagnostic work-up of T-cell clonality by FCM, particularly in the absence of phenotypic aberrations and when suspicious effector (e.g., LGL) Tαβ-cell populations are investigated. Further analysis including larger series of samples are required to establish the precise TRBC1^+^/TRBC1^−^ ratios within the different maturation compartments of Tαβ-cells, both in blood and in other tissues. 

Validation of the pre-established normal cut-offs in a large cohort of samples, including HD, reactive lymphocytosis and T-CLPD patients, showed a high degree of correlation between the TRBC1-FCM approach and T-cell clonality results assessed by molecular and TCRVβ-FCM assays, except for a few discrepant cases, in line with previous observations [18,21,25]. More detailed investigation of such discrepant cases showed that in three of five patients (cases #1, #2 and #4), final diagnosis was concordant with the TRBC1-based FCM assay results, with failure of PCR in two of three cases (cases #1 and #2) to detect T-cell clonality being due to the analysis of whole blood DNA instead of the FACS-sorted (suspicious) cell population DNA, in samples where the percentage of clonal cells in PB was below the sensitivity limit of the molecular technique [15,16,17]. Although final diagnosis at the referring center was inconclusive in the first case (case #1), the presence of a clearly aberrant (CD2^lo^) Tαβ effector cell population associated with chronic neutropenia would strongly support the diagnosis of T-LGLL [33], in line with monotypic expression of TRBC1. In one of the remaining two discrepant cases (case #3), an expansion of LGL with a normal Tαβ-effector cell phenotype (CD2^+^ CD7^lo^ CD27^−^ CD28^−^ CD45RA^−/+^ cytoplasmic granzyme^+^) [33], but an elevated TRBC1^+^/TRBC1^−^ ratio compared to normal/reactive polyclonal TαβCD8^+^ cells was observed in the absence of clonality by PCR. In this case, the possibility that the altered TRBC1^+^/TRBC1^−^ ratio might be due to an increased number of (activated-oligoclonal) senescent effector memory/terminal effector TαβCD8^+^ cells [14,15] could not be ruled out, since similarly increased TRBC1^+^/TRBC1^−^ ratios were observed in normal and reactive blood when analysis was restricted to normal effector memory/terminal effector TαβCD8^+^ cells. Finally, in the last discrepant case (case #5), an aberrant (CD2^lo^ CD7^-/lo^) terminal-effector (CD28^−^ CD45RA^+^ CD45RO^−^) phenotype suggestive of a monoclonal expansion, confirmed by PCR (but not by TCRVβ-FCM analysis and the new TRBC1-FCM assay) was observed in the absence of a definitive explanation for the discordant results.

Despite the low number of T-CLPD cases analyzed within each WHO2017 diagnostic category, our results showed a potentially skewed usage of TRBC2 vs. TRBC1 in T-PLL (derived from a naïve/central memory cell [1]), that contrast with PCTCL-SS (derived from a central memory/transitional memory cell [1]) and HDc (in whom most clones corresponded to effector memory/terminal effector), which more frequently involved the TRBC1 region. Our results support previous studies in which only one out of three T-PLL cases studied were TRBC1^+^ [21], while differing from others, in which only 22% of HDc (14/63, named as T-cell clones of uncertain significance) were TRBC1^+^ [23] or less than 50% of SS plus MF cases (30 of 63) showed TRBC1^+^ [21,24]. More extensive studies including larger series of a broader number of T-CLPD diagnostic categories are required to elucidate the potentially skewed usage of TRBC1 vs. TRBC2 in different diagnostic subtypes of T-CLPD, and the precise biological significance of these findings. 

Despite the diagnostic value of the new TRBC1 assay here optimized and validated, it should be noted that occasionally clonal T-cell populations show dim or negative surface CD3/TCRVβ expression levels [26]. Among those T-CLPD patients with productive gene rearrangements upstream of TRBC1, expression of TRBC1 at the protein level would be also low or negative. Thus, absence of TRBC1 expression in these patients would be due to lack of surface CD3/TRBC1 expression rather than a gene rearrangement upstream of TRBC2. In this regard, it should be noted that in nearly half of our TRBC1^+^ T-CLPD patients concordantly low expression of SmCD3 and TRBC1 was observed. In contrast, in the other half of our T-CLPD cases, clonal Tαβ-cells showed normal expression levels of SmCD3/TCRVβ with abnormally low amounts of TRBC1 on the cell surface membrane, in the absence of an apparent cause that could explain such differences, as previously reported by other research groups [23,25,26]. 

As described above, assessment of the TRBC1 expression profile among phenotypically aberrant populations of Tαβ-cells allowed sensitive identification of small Tαβ-cell clones, even among otherwise healthy donors, in line with previous findings [20,23,24,25]. However, the precise analytical sensitivity of the new TRBC1 FMC assay remained to be established. Thus, an additional objective of our study was to determine the level of detection of the TRBC1-based FCM approach for detecting clonal populations of Tαβ-cells showing an aberrant phenotype, whenever present at low frequencies in blood. From a clinical point of view, a level of detection of 10^−4^ is currently considered sufficient for minimal/measurable (residual) disease (MRD) detection and monitoring in T-CLPD [27,47]. In this regard, here we show that assessment of the pattern of expression of TRBC1 in small populations of phenotypically aberrant Tαβ-cells from patients with distinct subtypes of T-CLPD provides a level of detection of ≤10^−4^, similar to that obtained once tumor-associated TCRVβ family-specific antibodies are used, as demonstrated by real and virtual dilutional experiments. However, combined assessment of both TRBC1 and TCRVβ family expression profiles slightly improved the sensitivity of detection of small Tαβ-cell clones in one of our cases, and at the same time it provided more accurate MRD counts.

## 5. Conclusions

In summary, the here optimized TRBC1 approach is a useful, simple and fast FCM assay for assessment of Tαβ-cell clonality in blood of patients suspicious of T-CLPD. At the same time, the assay is cost-effective, since we have estimated an overall cost-saving of around 80–90% per sample compared to using either the whole TCRVβ Repertoire FCM Kit or PCR-based techniques. In addition, once used in combination with tumor-associated aberrant immunophenotypes, the TRBC1 expression profile (negative vs. positive) of SmCD3^+^ cells shows a high sensitivity and specificity for detection of monoclonal Tαβ-cells in patients suspicious of T-CLPD, at similar levels to those reached with the kappa/lambda ratio in the diagnostic work-up of B-CLPD [43,44,48]. Optimal assessment of clonality by TRBC1 expression would require appropriate integration of the TRBC1/CD3 reagents into comprehensive lymphocyte screening panels for the diagnostic work-up of patients presenting with lymphocytosis, as well as into the current T-CLPD classification and MRD monitoring antibody panels.

## Figures and Tables

**Table 1:** Number of T-CLPD cases (*n* = 87), analyzed according to their WHO2017 diagnosis.

Diagnosis	*n*. of Cases
T-PLL	10
PCTCL-SS	16
PCTCL-MF	6
PCTCL-NOS	1
PTCL-AITL	2
Extranodal NK/T-lymphoma, nasal type	1
PCTCLPD-small/medium CD4	2
Hemophagocytic syndrome	1
PTCL-NOS	2
T-LGLL	40
T-CLPD not classified	6

Abbreviations (alphabetical order): AITL, angioimmunoblastic T-cell lymphoma; CLPD, chronic lymphoproliferative disorder; MF, mycosis fungoides; *n*., number; NOS, not otherwise specified; PCTCL, primary cutaneous T-cell lymphoma; PCTCLPD, primary cutaneous T chronic lymphoproliferative disorder; PTCL, peripheral T-cell lymphoma; SS, Sézary syndrome; T-LGLL, T-cell large granular lymphocyte leukemia; T-PLL, T-cell prolymphocytic leukemia; WHO, World Health Organization.

**Table 2:** TRBJ gene rearrangements of FACS-sorted TRBC1 positive and/or TRBC1 negative Tαβ^+^ cell populations (*n* = 95).

TRBC1 Expression by FCM	Clonality Status of TRBC1 Stained Cell Populations ^1^	TRBJ Rearrangement	
		JB1	JB1+JB2
Positive (*n* = 47)	Monoclonal (*n* = 4)	4	0
	Oligoclonal (*n* = 3)	3	0
	Polyclonal (*n* = 40)	37	3
	TOTAL	44/47 (94%)	3/47 (6%)
Negative (*n* = 48)	Monoclonal (*n* = 3 ^2^)	0	3
	Oligoclonal (*n* = 4)	0	4
	Polyclonal (*n* = 41)	0	41
	TOTAL	0	48/48 (100%)

All FACS-sorted cell populations (purity ≥ 95%) showed unequivocal expression of CD3 on the cell surface membrane by FCM with optimal PCR amplification of the TRBJ gene product. Monoclonal populations were isolated from three HDc and four T-CLPD patients; oligoclonal populations from three reactive lymphocytosis, two HDc and one T-CLPD patient; and polyclonal populations from 13 HD, seven reactive lymphocytosis, four HDc and two T-CLPD patients. ^1^ The clonal nature (mono vs. oligo vs. polyclonal) of each purified cell population was assessed by TRB gene rearrangement analysis. ^2^ One cell population had CD3^high^ expression. Abbreviations (alphabetical order): FCM, flow cytometry; N, number; PCR, polymerase chain region; TR, T-cell receptor.

**Table 3:** Ranges for polyclonal (normal and reactive) total Tαβ-cells and their major Tαβ-cell populations in PB (*n* = 83) as defined by the mean percentage of TRBC1^+^ cells and the mean TRBC1^+^/TRBC1^−^ ratio ± 3 standard deviations (3 SD).

Tαβ-Cell Subset	% TRBC1^+^ Cells *		TRBC1^+^/TRBC1^−^ Ratio		Probability (%) of Finding A Clonal Tαβ Expansion When TRBC1^+^/TRBC1^−^ Ratio is Outside the Range Mean ± 3 SD (*ρ*-Value)
	Mean ± 1 SD	Range (Mean ± 3 SD)	Mean ± 1 SD	Range (Mean ± 3 SD)	
Tαβ cells	40 ± 6.7	20–60	0.66 ± 0.071	0.25–1.4	99.73% (<0.001)
Tαβ CD4^+^	43 ± 6.3	24–62	0.75 ± 0.067	0.31–1.6	
Tαβ CD8^+^	35 ± 8.8	8.3–61	0.53 ± 0.096	0.091–1.6	
Tαβ DP	36 ± 12	1.6–71	0.57 ± 0.13	0.016–2.5	
Tαβ DN	29 ± 10	0-61	0.41 ± 0.12	0–1.5	

* Conventional normality tests confirmed that this variable showed a Gaussian distribution. Abbreviations (alphabetical order): DN, doble negative; DP, double positive; PB, peripheral blood; SD, standard deviation; TR, T-cell receptor.

**Table 4:** Comparison between TRBC1 assay by FCM and the reference molecular and FCM techniques used to assess Tαβ-cell clonality (*n* = 117).

Clonality Status by Other Techniques *	TRBC1 Expression Pattern by FCM		*p*-Value
	Polytypic (*n* = 23)	Monotypic (*n* = 94)	
Poly/oligoclonal (*n* = 24)	**21/24 (87%)**	3/24 (13%)	<0.0001
Monoclonal (*n* = 93)	2/93 (2%)	**91/93 (98%)**	

Concordant cases are highlighted in bold. * Clonality assessed by PCR, TCRVβ family expression by FCM and/or gene mutation assays. Abbreviation: FCM, flow cytometry; PCR, polymerase chain region; TR, T-cell receptor.

**Table 5:** TRBC1 expression profile in different WHO diagnostic categories of SmCD3^+^ Tαβ-cell CLPD (*n* = 79) plus HDc (*n* = 10).

WHO 2017 Diagnosis	TRBC1^+^ (*n* = 52)	TRBC1^−^ (*n* = 37)
T-PLL (*n* = 10)	3/10 (30%)	7/10 (70%)
PCTCL-SS (*n* = 15)	12/15 (80%)	3/15 (20%)
PCTCL-MF (*n* = 6)	3/6 (50%)	3/6 (50%)
PCTCL-NOS (*n* = 1)	0/1 (0%)	1/1 (100%)
PTCL-AITL (*n* = 2)	0/2 (0%)	2/2 (100%)
Extranodal NK/T-lymphoma, nasal type (*n* = 1)	0/1 (0%)	1/1 (100%)
PCTCLPD-small/medium CD4 (*n* = 2)	2/2 (100%)	0/2 (0%)
Hemophagocytic syndrome (*n* = 1)	0/1 (0%)	1/1 (100%)
PTCL-NOS (*n* = 2)	1/2 (50%)	1/2 (50%)
T-LGLL (*n* = 39)	22/39 (56%)	17/39 (44%)
HDc * (*n* = 10)	9/10 (90%)	1/10 (10%)

14/52 (27%) of TRBC1 positive cases were TRBC1^lo^, and 6/14 (43%) of TRBC1^lo^ cases were CD3^lo^. * Information about the expression profile of TRBC1 is referred to clonal cells. Abbreviations (alphabetical order): AITL, angioimmunoblastic T-cell lymphoma; CLPD, chronic lymphoproliferative disorders; HD, healthy donor; HDc, healthy donor with a small Tαβ-cell clone in blood; MF, mycosis fungoides; NOS, not otherwise specified; PCTCL, primary cutaneous T-cell lymphomas; PCTCLPD, primary cutaneous T chronic lymphoproliferative disorder; PTCL, peripheral T-cell lymphoma; Sm, surface membrane; SS, Sézary syndrome; T-LGLL, T-cell large granular lymphocyte leukemia; T-PLL, T-cell prolymphocytic leukemia; TR, T-cell receptor; WHO, World Health Organization.

## Data Availability

Exclude this statement as the study did not report any data.

## Supplementary Materials

The following are available online at https://www.mdpi.com/article/10.3390/cancers13174379/s1. Supplementary Methods. Table S1: Fluorochrome-conjugated antibody panels used in this study. Table S2: Sources and specificities of the monoclonal antibody reagents used. Table S3: Phenotypic markers associated with different maturation subsets of T-cells and the corresponding immunophenotypic profiles used for the identification of T-cell maturation stages in adult healthy donor blood. Table S4: Distribution of TRBC1^+^ cells and the TRBC1^+^/TRBC1^−^ T-cell ratio among normal and reactive Tαβ-cells and the major Tαβ-cell populations present in normal and reactive PB. Table S5: Detailed features of samples showing discrepant results between the pattern of expression of TRBC1 by FCM and the T-cell clonality status as assessed by the reference molecular and TCRVβ-FCM assays (*n* = 5/117; 4.3%). Figure S1: Flowchart illustrating the distribution of samples (*n* = 211, including 192 PB, 9 SK, 5 BM, 4 LN and 1 AM) and the study groups corresponding to the different sets of experiments performed. Figure S2: Pattern of expression of TRBC1 on blood T-cells using different staining conditions. Figure S3: TRBC1^+^/TRBC1^−^ ratio of normal Tαβ-cell subsets defined within the major TCD4, TCD8, TDP and TDN cell populations based on the pattern of expression of specific TCRVβ family members and their maturation stage. Figure S4: Illustrative dot-plot diagrams, clonality data and final diagnosis of cases showing discrepant results between the pattern of expression of TRBC1 by FCM and the T-cell clonality status by conventional TCRVβ-FCM and/or molecular reference techniques (*n* = 5/117; 4.3%). Figure S5: Illustrative dot-plot diagrams of a representative dilutional experiment of clonal Tαβ-cells from a T-LGLL patient in normal blood.
