# Supplementary material for: Anti-TRBC1 Antibody-Based Flow Cytometric Detection of T-Cell Clonality: Standardization of Sample Preparation and Diagnostic Implementation
Source: Cancers (Basel). 2021 Aug 30;13(17):4379. doi: 10.3390/cancers13174379 (PMC8430560; doi:10.3390/cancers13174379)
Supplement: Supplementary file 1 [file cancers-13-04379-s001.zip › cancers-1320004-supplementary.pdf]

cancers

MDPI

**Supplementary Material**

## 1. Supplementary Methods

### *1.1. Patients, Controls and Samples.*

The distribution of samples and study groups included in each of the sets of experiments performed was as follows: peripheral blood (PB) samples from 33 healthy donors (HD) were first used for optimization of the TRBC1 staining procedure (including reagent titration) for flow cytometry (FCM). In turn, 26 PB and 2 bone marrow (BM) samples from 13 HD, 7 patients with reactive lymphocytosis, 4 HD with small T-cell clone in blood (HDc) and 4 patients with T-cell chronic lymphoproliferative disorders (T-CLPD) were used to analyze TRBJ1 vs. TRBJ2 gene rearrangements by PCR on FACS-sorted TRBC1$^+$ and TRBC1$^-$ Tαβ-cell populations (Figure S1). Normal/reactive PB ranges for the percentage of TRBC1$^+$ cells (and the TRBC1$^+$/TRBC1$^-$ ratio) within the different major subsets of Tαβ-cells—i.e., TαβCD4$^+$, TαβCD8$^+$, Tαβ double-positive (DP) and Tαβ double-negative (DN) cells—were derived from a total of 83 PB samples from HD ($n$ = 65), and subjects with reactive lymphocytosis ($n$ = 18) (Figure S1). In addition, a subset of 27 PB samples from HD ($n$ = 12), subjects with reactive lymphocytosis ($n$ = 10) and HDc ($n$ = 5) plus 10 PB samples from HD, were further used to define normal TRBC1$^+$/TRBC1$^-$ ratio ranges within the different T-cell subsets defined by the expression of 24 different TCRVβ families and T-cell maturation stages, respectively (Figure S1). The degree of agreement between clonality status by the TRBC1-based FCM assay and conventional molecular and FCM techniques, was evaluated in 117 samples (2 HD, 21 reactive lymphocytosis, 7 HDc and 87 T-CLPD) containing either poly(oligo)clonal and/or monoclonal Tαβ-cells. Finally, analysis of TRBC1 expression (either positive or negative) on clonal T-cells from a total of 79 Tαβ-CLPD diagnosed according to the World Health Organization (WHO) 2017 criteria and 10 HDc, was performed (Figure S1). To determine the sensitivity of the anti-TRBC1 antibody reagent for detecting clonal Tαβ-cells, dilutional experiments ($n$ = 8) of clonal PB T-cells from 5 T-CLPD patients and 1 HDc in normal HD PB specimens (2 real and 6 in silico), were performed (Figure S1).

### *1.2. Optimization of TRBC1 Staining for Flow Cytometry.*

Competition assays with distinct purified (SK7 or UCHT1 clones) and fluorochrome-conjugated CD3 reagent clones (SK7, REA613 and UCHT1) were performed in paired aliquots of 6 PB samples from HD (Table S1A). All reagents were tested under three different incubation conditions where: a) the conjugated reagent was added first, followed by a 10 min incubation after which the unconjugated antibody reagent was added; b) the conjugated and unconjugated antibody reagents were added simultaneously; and c) the unconjugated reagent was added first, 10 min before the fluorochrome-conjugated reagent.

The potential (steric) interaction between surface membrane CD3 and TRBC1 was subsequently tested in paired aliquots of 11 HD PB samples under four different staining conditions: a) staining with TRBC1 only; and with both CD3 and anti-TRBC1 reagents where CD3 was added b) 10 min after the anti-TRBC1 reagent; c) simultaneously; or d) 10 min before TRCB1. Subsequently, different fluorochrome-conjugated anti-TRBC1 reagents—fluorescein-5-isothiocyanate (FITC) and Brilliant Violet (BV)421 conjugated reagents—of the TRBC1 JOVI-1 clone and CD3 reagents—i.e., SK7-allophycocyanin (APC), SK7-phycoerythrin (PE) cyanin 7 (Cy7), REA613-APC, REA613-PEVio770 and UCHT1-PECF594 clone-fluorochrome reagents—were tested in the presence of anti-CD4, CD8, CD19, CD45 and TCRγδ monoclonal antibodies (Mab) (Table S1B). Titration of the different anti-TRBC1 antibody reagents was performed in 2 PB samples, by adding the anti-TRBC1 reagent simultaneously and/or 10 min after CD3.

In a following step, simultaneous staining with different combination of CD3 i.e., different CD3 clone-fluorochrome conjugates (SK7-PerCPCy5.5, REA613-PEVio770 and

SK7-APC) and anti-TRBC1 (clone JOVI-1) reagent conjugates (BV421 and BV786), were compared for one vs. two washes in phosphate-buffered saline (PBS) containing 0.5% bovine serum albumin (BSA) (540 g for 5 min) after the red cell lysing step (Table S1C).

Finally, two samples were stained with FITC- and BV421-conjugated anti-TRBC1 reagents plus CD3 (added simultaneously) immediately after sample collection and subsequently at 24 h, 48 h and 72 h (Table S1D). For the later time points, samples were kept at 4 ºC until stained.

The steric interaction between TCRVβ and TRBC1 was also evaluated in 2 PB samples from HD for each of the 24 TCRVβ families included in the IOTest® Beta Mark TCRVβ Repertoire Kit (Beckman Coulter, Brea, CA) under three different incubation conditions: a) addition of TRBC1, followed by a 10 min incubation before the TCRVβ reagents were added; b) simultaneous addition of TRBC1 and the TCRVβ reagents; and c) addition of the TCRVβ reagents 10 min before TRBC1 (Table S1E). In all these experiments, CD3 was added 10 min after the TRBC1 plus TCRVβ reagents had been stained.

**Table S1.** Fluorochrome-conjugated antibody panels used in this study.

**Suppl. Panel A. Competition assays between purified and fluorochrome-conjugated CD3 reagents**

*Suppl. Panel A.1. Purified SK7 or UCHT1 clones of the CD3 reagent vs. APC-conjugated CD3 (clone SK7)*

| Staining conditions | PacB | OC515 | FITC | APC | APCH7 |
|---|---|---|---|---|---|
| Conjugated 10′ and then purified | CD4 | CD45 | CD16 | SmCD3 | CD19 |
| Purified + Conjugated | CD4 | CD45 | CD16 | SmCD3 | CD19 |
| Purified 10′ and then conjugated | CD4 | CD45 | CD16 | SmCD3 | CD19 |

*Suppl. Panel A.2. Purified SK7 or UCHT1 clones of the CD3 reagent vs. APC-conjugated CD3 (clone REA613)*

| Staining conditions | PacB | OC515 | FITC | PEVio770 | APCH7 |
|---|---|---|---|---|---|
| Conjugated 10′ and then purified | CD4 | CD45 | CD16 | SmCD3 | CD19 |
| Purified + Conjugated | CD4 | CD45 | CD16 | SmCD3 | CD19 |
| Purified 10′ and then conjugated | CD4 | CD45 | CD16 | SmCD3 | CD19 |

*Suppl. Panel A.3. Purified SK7 or UCHT1 clones of the CD3 reagent vs. APC-conjugated CD3 (clone UCHT1)*

| Staining conditions | PacB | OC515 | FITC | PerCPCy5.5 | APCH7 |
|---|---|---|---|---|---|
| Conjugated 10′ and then pure | SmCD3 | CD45 | CD16 | CD4 | CD19 |
| Purified + Conjugated | SmCD3 | CD45 | CD16 | CD4 | CD19 |
| Purified 10′ and then conjugated | SmCD3 | CD45 | CD16 | CD4 | CD19 |

**Suppl. Panel B. Staining conditions for evaluation of the pattern of expression of CD3 vs. TRBC1**

*Suppl. Panel B.1. APC-conjugated CD3 reagent (clone SK7) and FITC-conjugated TRBC1 reagent (clone JOVI-1)*

| Staining conditions | PacB | OC515 | FITC | PECy7 | APC | APCH7 |
|---|---|---|---|---|---|---|
| TRBC1 only | CD4 | CD45 | TRBC1 | TCRγδ | - | CD8 |
| TRBC1 10′ and then CD3 | CD4 | CD45 | TRBC1 | TCRγδ | SmCD3 | CD8 |
| CD3 + TRBC1 | CD4 | CD45 | TRBC1 | TCRγδ | SmCD3 | CD8 |
| CD3 10′ and then TRBC1 | CD4 | CD45 | TRBC1 | TCRγδ | SmCD3 | CD8 |

*Suppl. Panel B.2. APC-conjugated CD3 reagent (clone SK7) and BV421-conjugated TRBC1 reagent (clone JOVI-1)*

| Staining conditions | BV421 | OC515 | PerCPCy5.5 | PECy7 | APC | APCH7 |
|---|---|---|---|---|---|---|
| TRBC1 only | TRBC1 | CD45 | CD4 | CD19 + TCRγδ | - | CD8 |
| TRBC1 10′ and then CD3 | TRBC1 | CD45 | CD4 | CD19 + TCRγδ | SmCD3 | CD8 |
| CD3 + TRBC1 | TRBC1 | CD45 | CD4 | CD19 + TCRγδ | SmCD3 | CD8 |
| CD3 10′ and then TRBC1 | TRBC1 | CD45 | CD4 | CD19 + TCRγδ | SmCD3 | CD8 |

*Suppl. Panel B.3. PECy7-conjugated CD3 reagent (clone SK7) and FITC-conjugated TRBC1 reagent (clone JOVI-1)*

| Staining conditions | FITC | PerCPCy5.5 | PECy7 | APC | AF700 | APCH7 |
|---|---|---|---|---|---|---|
| TRBC1 only | TRBC1 | CD8 | - | TCRγδ | CD45 | CD4 |
| TRBC1 10′ and then CD3 | TRBC1 | CD8 | SmCD3 | TCRγδ | CD45 | CD4 |
| CD3 + TRBC1 | TRBC1 | CD8 | SmCD3 | TCRγδ | CD45 | CD4 |
| CD3 10′ and then TRBC1 | TRBC1 | CD8 | SmCD3 | TCRγδ | CD45 | CD4 |

*Suppl. Panel B.4. PECy7-conjugated CD3 reagent (clone SK7) and BV421-conjugated TRBC1 reagent (clone JOVI-1)*

| Staining conditions | BV421 | OC515 | FITC | PerCPCy5.5 | PEVio770 | APC | APCH7 |
|---|---|---|---|---|---|---|---|
| TRBC1 only | TRBC1 | CD45 | TCRγδ | CD4 | - | CD19 | CD8 |
| TRBC1 10′ and then CD3 | TRBC1 | CD45 | TCRγδ | CD4 | SmCD3 | CD19 | CD8 |
| CD3 + TRBC1 | TRBC1 | CD45 | TCRγδ | CD4 | SmCD3 | CD19 | CD8 |
| CD3 10′ and then TRBC1 | TRBC1 | CD45 | TCRγδ | CD4 | SmCD3 | CD19 | CD8 |

*Suppl. Panel B.5. APC-conjugated CD3 reagent (clone REA613) and FITC- or BV421-conjugated TRBC1 reagent (clone JOVI-1)*

| Staining conditions | FITC/BV421 | OC515 | PerCPCy5.5 | PECy7 | APC | APCH7 |
|---|---|---|---|---|---|---|
| TRBC1 only | TRBC1 | CD45 | CD4 | TCRγδ | - | CD8 |
| TRBC1 10′ and then CD3 | TRBC1 | CD45 | CD4 | TCRγδ | SmCD3 | CD8 |
| CD3 + TRBC1 | TRBC1 | CD45 | CD4 | TCRγδ | SmCD3 | CD8 |
| CD3 10′ and then TRBC1 | TRBC1 | CD45 | CD4 | TCRγδ | SmCD3 | CD8 |

*Suppl. Panel B.6. PEVio770-conjugated CD3 reagent (clone REA613) and FITC-conjugated TRBC1 reagent (clone JOVI-1)*

| Staining conditions | FITC | PerCPCy5.5 | PEVio770 | APC | AF700 | APCH7 |
|---|---|---|---|---|---|---|
| TRBC1 only | TRBC1 | CD8 | - | TCRγδ | CD45 | CD4 |
| TRBC1 10′ and then CD3 | TRBC1 | CD8 | SmCD3 | TCRγδ | CD45 | CD4 |
| CD3 + TRBC1 | TRBC1 | CD8 | SmCD3 | TCRγδ | CD45 | CD4 |
| CD3 10′ and then TRBC1 | TRBC1 | CD8 | SmCD3 | TCRγδ | CD45 | CD4 |

*Suppl. Panel B.7. PEVio770-conjugated CD3 reagent (clone REA613) and BV421-conjugated TRBC1 reagent (clone JOVI-1)*

| Staining conditions | BV421 | OC515 | FITC | PerCPCy5.5 | PEVio770 | APC | APCH7 |
|---|---|---|---|---|---|---|---|
| TRBC1 only | TRBC1 | CD45 | TCRγδ | CD4 | - | CD19 | CD8 |
| TRBC1 10′ and then CD3 | TRBC1 | CD45 | TCRγδ | CD4 | SmCD3 | CD19 | CD8 |
| CD3 + TRBC1 | TRBC1 | CD45 | TCRγδ | CD4 | SmCD3 | CD19 | CD8 |
| CD3 10′ and then TRBC1 | TRBC1 | CD45 | TCRγδ | CD4 | SmCD3 | CD19 | CD8 |

*Suppl. Panel B.8. PECF594-conjugated CD3 reagent (clone UCHT1) and FITC- or BV421-conjugated TRBC1 reagent (clone JOVI-1)*

| Staining conditions | BV421 | PerCPCy5.5 | PECF594 | APC | AF700 | APCH7 |
|---|---|---|---|---|---|---|
| TRBC1 only | TRBC1 | CD8 | - | TCRγδ | CD45 | CD4 |
| TRBC1 10′ and then CD3 | TRBC1 | CD8 | SmCD3 | TCRγδ | CD45 | CD4 |
| CD3 + TRBC1 | TRBC1 | CD8 | SmCD3 | TCRγδ | CD45 | CD4 |
| CD3 10′ and then TRBC1 | TRBC1 | CD8 | SmCD3 | TCRγδ | CD45 | CD4 |

**Suppl. Panel C. Comparison of performing 1 vs. 2 washing steps after red cell lysis**

| Tube | CD3 fluorochrome | BV421 | OC515 | BV786 | FITC | PerCPCy5.5 | PECy7/PEVio770 | APC | APCH7 |
|---|---|---|---|---|---|---|---|---|---|
| 1 | PerCPCy5.5 | TRBC1 | CD45 | - | - | SmCD3 | CD19 + TCRγδ | CD8 | CD4 |
| 2 | PerCPCy5.5 | - | CD45 | TRBC1 | - | SmCD3 | CD19 + TCRγδ | CD8 | CD4 |
| 3 | APC | TRBC1 | CD45 | - | - | CD4 | CD19 + TCRγδ | SmCD3 | CD8 |
| 4 | APC | - | CD45 | TRBC1 | - | CD4 | CD19 + TCRγδ | SmCD3 | CD8 |
| 5 | PEVio770 | TRBC1 | CD45 | - | TCRγδ | CD4 | SmCD3 | CD19 | CD8 |
| 6 | PEVio770 | - | CD45 | TRBC1 | TCRγδ | CD4 | SmCD3 | CD19 | CD8 |

**Suppl. Panel D. Comparison of different times of incubation with the TRBC1 reagent (at 0, 24, 48, 72 h)**

| BV421 | OC515 | PerCPCy5.5 | PECy7 | APC | APCH7 | OC515 | FITC | PerCPCy5.5 | PECy7 | APC | APCH7 |
|---|---|---|---|---|---|---|---|---|---|---|---|
| TRBC1 | CD45 | CD4 | TCRγδ | SmCD3 | CD8 | CD45 | TRBC1 | CD4 | TCRγδ | SmCD 3 | CD8 |

**Suppl. Panel E. Incubation conditions for simultaneous staining with TCRVβ and TRBC1**

| Staining conditions | PacB | OC515 | FITC | PE | PerCPCy5.5 | PECy7 | Dy634 | APCH7 |
|---|---|---|---|---|---|---|---|---|
| TRBC1 10′ and then TCRVβ | CD4 | CD45 | TCR-Vβ A | TCR-Vβ B | CD3 | TCRγδ | TRBC1 | CD8 |
| Simultaneous incubation | CD4 | CD45 | TCR-Vβ A | TCR-Vβ B | CD3 | TCRγδ | TRBC1 | CD8 |
| TCRVβ 10′ and then TRBC1 | CD4 | CD45 | TCR-Vβ A | TCR-Vβ B | CD3 | TCRγδ | TRBC1 | CD8 |

| Suppl. Panel F. Simultaneous staining with the IOTest® Beta Mark TCR-Vβ Repertoire Kit (Beckman Coulter) and TRBC1 | | | | | | | |
|---|---|---|---|---|---|---|---|
| **PacB/BV421** | **BV480/OC515** | **FITC** | **PE** | **PerCPCy5.5** | **PECy7** | **APC/Dy634** | **APCH7** |
| CD45 | TRBC1 | TCR-Vβ A | TCR-Vβ B | CD4 | TCRγδ | SmCD3 | CD8 |
| TRBC1 | CD45 | TCR-Vβ A | TCR-Vβ B | CD4 | TCRγδ | SmCD3 | CD8 |
| CD4 | CD45 | TCR-Vβ A | TCR-Vβ B | CD3 | TCRγδ | TRBC1 | CD8 |

| Suppl. Panel G. Analysis of TRBC1 expression on different maturation-associated subsets of Tαβ-cells | | | | | | | | | | | | | |
|---|---|---|---|---|---|---|---|---|---|---|---|---|---|
| **BV421** | **BV510** | **BV605** | **BV650** | **BV711** | **BV786** | **FITC** | **PerCP Cy5.5** | **PECF 594** | **PE** | **PECy7** | **APC** | **AF700** | **APC H7** |
| CD27 | CD45RA | CD62L | CD25 | CD127 | CD3 | TRBC1 | CD28 | CD8 | cyGra | TCRγδ | CD57 | CD45 | CD4 |

For all tubes, "stain & lyse" EuroFlow SOPs were used (www.EuroFlow.com) [29], with the modifications described above. Abbreviations (alphabetical order): AF700, Alexa Fluor®700; APC, allophycocyanin; APCH7, APC Hilite®7; BV, Brilliant Violet™; cy, cytoplasmic; Dy, dyomics; FITC, fluorescein isothiocyanate; Gra, granzyme B; h, hours; OC515, Orange Cytognos™ 515; PacB, Pacific Blue™; PE, phycoerythrin; PECy7, phycoerythrin-cyanin 7; PerCPCy5.5, peridinin-chlorophyll-cyanin 5.5; Sm, surface membrane; TCR, T-cell receptor.

**Table S2.** Sources and specificities of the monoclonal antibody reagents used.

| Marker | Fluorochrome | Clone | Manufacturer | Volume (μL) |
|---|---|---|---|---|
| CD3 | Pure | SK7 | BD | 20 |
| CD3 | Pure | UCHT1 | Immunostep | 2 |
| CD3 | APC | SK7 | BD | 5 |
| CD3 | BV786 | SK7 | BD | 1 |
| CD3 | PacB | UCHT1 | BD | 5 |
| CD3 | PECy7 | SK7 | BD | 5 |
| CD3 | PEVio770 | REA613 | Miltenyi | 5 |
| CD3 | PerCPCy5.5 | SK7 | BD | 10 |
| CD3 | PECF594 | UCHT1 | BD | 5 |
| CD4 | APCH7 | SK3 | BD | 5 |
| CD4 | PacB | RPA-T4 | BioLegend | 2 |
| CD4 | PerCPCy5.5 | SK3 | BD | 10 |
| CD8 | APC | SK1 | BD | 5 |
| CD8 | APCH7 | SK1 | BD | 5 |
| CD8 | PECF594 | RPA-T8 | BD | 1 |
| CD8 | PerCPCy5.5 | SK1 | BD | 10 |
| CD16 | FITC | 3G8 | Beckman Coulter | 10 |
| CD19 | APC | 5525C1 | BD | 5 |
| CD19 | APCH7 | SJ25C1 | BD | 5 |
| CD19 | PECy7 | J3119 | Immunostep | 5 |
| CD25 | BV650 | M-A251 | BD | 5 |
| CD27 | BV421 | MT271 | BD | 2 |
| CD28 | PerCPCy5.5 | CD28.2 | BioLegend | 5 |
| CD45 | AF700 | HI30 | BD | 2.5 |
| CD45 | OC515 | GA90 | Cytognos | 5 |
| CD45 | PacB | T26/33 | Dako | 5 |
| CD45RA | BV510 | HI100 | BD | 2.5 |
| CD57 | APC | NK-1 | BD | 1 |
| CD62L | BV605 | DREG56 | BioLegend | 2.5 |
| CD127 | BV711 | HIL7RM21 | BD | 5 |
| Granzyme B | PE | GB11 | Sanquin | 5 |
| TCRγδ | APC | B1 | BD | 5 |
| TCRγδ | FITC | IMMU510 | Beckman Coulter | 10 |
| TCRγδ | PECy7 | 11F2 | BD | 1 |

| | | | | |
|---|---|---|---|---|
| TRBC1 | BV421 | JOVI-1 | BD | 2 |
| TRBC1 | BV480 | JOVI-1 | BD | 2 |
| TRBC1 | BV650 | JOVI-1 | BD | 2 |
| TRBC1 | BV786 | JOVI-1 | BD | 2 |
| TRBC1 | Dy634 | JOVI-1 | Immunostep | 0.2 |
| TRBC1 | FITC | JOVI-1 | Immunostep | 1.6 |
| TCRVβ 5.3 | PE | 3D11 | Beckman Coulter | |
| TCRVβ 7.1 | PE + FITC | ZOE | Beckman Coulter | 10 (Tube A) |
| TCRVβ 3 | FITC | CH92 | Beckman Coulter | |
| TCRVβ 9 | PE | FIN9 | Beckman Coulter | |
| TCRVβ 17 | PE + FITC | E17.5F3 | Beckman Coulter | 10 (Tube B) |
| TCRVβ 16 | FITC | TAMAYA1.2 | Beckman Coulter | |
| TCRVβ 18 | PE | BA62.6 | Beckman Coulter | |
| TCRVβ 5.1 | PE + FITC | IMMU157 | Beckman Coulter | 10 (Tube C) |
| TCRVβ 20 | FITC | ELL1.4 | Beckman Coulter | |
| TCRVβ 13.1 | PE | IMMU222 | Beckman Coulter | |
| TCRVβ 13.6 | PE + FITC | JU74.3 | Beckman Coulter | 10 (Tube D) |
| TCRVβ 8 | FITC | 56C5.2 | Beckman Coulter | |
| TCRVβ 5.2 | PE | 36213 | Beckman Coulter | |
| TCRVβ 2 | PE + FITC | MPB2D5 | Beckman Coulter | 10 (Tube E) |
| TCRVβ 12 | FITC | VER2.32 | Beckman Coulter | |
| TCRVβ 23 | PE | AF23 | Beckman Coulter | |
| TCRVβ 1 | PE + FITC | BL37.2 | Beckman Coulter | 10 (Tube F) |
| TCRVβ 21.3 | FITC | IG125 | Beckman Coulter | |
| TCRVβ 11 | PE | C21 | Beckman Coulter | |
| TCR-Vβ 22 | PE + FITC | IMMU546 | Beckman Coulter | 10 (Tube G) |
| TCR-Vβ 14 | FITC | CAS1.1.3 | Beckman Coulter | |
| TCR-Vβ 13.2 | PE | H132 | Beckman Coulter | |
| TCR-Vβ 4 | PE + FITC | WJF24 | Beckman Coulter | 10 (Tube H) |
| TCR-Vβ 7.2 | FITC | ZIZOU4 | Beckman Coulter | |

Abbreviations (alphabetical order): AF700, Alexa Fluor®700; APC, allophycocyanin; APCH7, APC Hilite®7; BD, Becton/Dickinson Biosciences; BV, Brilliant Violet™; Dy, dyomics; FITC, fluorescein isothiocyanate; OC515, Orange Cytognos™ 515; PacB, Pacific Blue™; PE, phycoerythrin; PECy7, phycoerythrin-cyanin 7; PerCPCy5.5, peridinin-chlorophyll-cyanin 5.5; TCR, T-cell receptor.

**Table S3.** Phenotypic markers associated with different maturation subsets of T-cells and the corresponding immunophenotypic profiles used for the identification of T-cell maturation stages from adult healthy donor blood.

| T-cell maturation stages | CD25 | CD27 | CD28 | CD45RA | CD57 | CD62L | CD127 | cyGra |
|---|---|---|---|---|---|---|---|---|
| Treg* | ++ | | | | | | lo | |
| Naïve | - | + | + | + | - | + | + | - |
| CM | -/lo | + | + | - | - | + | + | - |
| TM | lo | + | + | - | | - | + | |
| CD28$^+$ EM | lo/- | - | + | - | | het | + | |
| CD28$^-$ EM | - | - | - | - | het | het | + | |
| EE | - | + | - | -/+ | het | het | + | |
| TE | - | - | - | + | het | het | + | + |

* Only within TαβCD4$^+$ cells. Abbreviations (alphabetical order): CM, central memory; cy, cytoplasmic; EE, early effector; EM, effector memory; Gra, granzyme B; het, heterogeneous; lo, low positive; TE, terminal effector; TM, transitional memory; Treg, regulatory T-cells.

**Table S4.** Distribution of TRBC1$^{+}$ cells and the TRBC1$^{+}$/TRBC1$^{-}$ T-cell ratio among normal and reactive Tαβ-cells and the major Tαβ-cell populations present in normal and reactive PB.

| **Tαβ cells and Tαβ-cell subsets** | **HD samples ($n$ = 65)** | | **Reactive lymphocytosis ($n$ = 18)** | |
|---|---|---|---|---|
| | % TRBC1$^{+}$ cells* | TRBC1$^{+}$/TRBC1$^{-}$ ratio | % TRBC1$^{+}$ cells* | TRBC1$^{+}$/TRBC1$^{-}$ ratio |
| Tαβ cells | 39 ± 5.8 | 0.63 ± 0.062 | 43 ± 8.5 | 0.75 ± 0.092 |
| Tαβ CD4$^{+}$ | 42 ± 5.9 | 0.72 ± 0.062 | 46 ± 7.0 | 0.84 ± 0.076 |
| Tαβ CD8$^{+}$ | 33 ± 7.5 | 0.50 ± 0.081 | 40 ± 11 | 0.67 ± 0.12 |
| Tαβ DP | 34 ± 11 | 0.51 ± 0.13 | 45 ± 8.2 | 0.81 ± 0.090 |
| Tαβ DN | 27 ± 10 | 0.38 ± 0.11 | 36 ± 8.5 | 0.57 ± 0.092 |

Results express as mean ± 1SD. * Conventional normality tests confirmed that this variable was normally distributed. Abbreviations (alphabetical order): DN, double negative; DP, double positive; SD, standard deviation. TR, T-cell receptor.

**Table S5.** Detailed features of samples showing discrepant results between the pattern of expression of TRBC1 by FCM and the T-cell clonality status as assessed by the reference molecular and TCRVβ-FCM assays ($n$ = 5/117; 4.3%).

| ***n*. case** | **Clonality status by the reference techniques** | | **TRBC1 pattern** (TRBC1$^{+}$/ TRBC1$^{-}$ ratio) | **Aberrant/pathological cells (by flow cytometry)** | | **Other features** | **Final Diagnosis** |
|---|---|---|---|---|---|---|---|
| | **Clonality** | **Technique** | | **Phenotype** | **% of leucocytes** | | |
| #1 | Polyclonal | PCR (whole PB) | Monotypic ( >99) | CD2$^{lo}$ CD3$^{lo}$ CD4$^{-}$ CD5$^{-/lo}$ CD7$^{+}$ CD8$^{+}$ CD11c$^{+}$ CD16$^{+}$ CD26$^{-}$ CD28$^{-}$ CD56$^{+}$ CD57$^{+}$ cyPerf$^{+}$ TRBC1$^{lo}$ | 2.5% | Chronic neutropenia | Inconclusive vs. T-LGLL |
| #2 | Polyclonal | PCR (whole PB) | Monotypic ( <0.01) | FSC/SSC$^{++}$ CD2$^{lo}$ CD3$^{lo}$ CD4$^{+}$ CD5$^{++}$ CD7$^{++}$ CD8$^{-}$ CD16$^{-}$ CD26$^{-}$ CD28$^{++}$ CD45$^{lo}$ CD56$^{-}$ CD57$^{-}$ TRBC1$^{-}$ | 5.9% | T-cell (mono)clonality by PCR in skin biopsy | PCTCL-SS |
| #3 | Oligoclonal | PCR (PB FACS-sorted population) | Monotypic (8) | CD2$^{+}$ CD3$^{lo}$ CD4$^{-}$ CD7$^{lo}$ CD8$^{++}$ CD16$^{-}$ CD27$^{-}$ CD28$^{-}$ CD45RA$^{-/+}$ CD45$^{++}$ CD56$^{-/+}$ CD57$^{+}$ CD62L$^{+}$ cyGra$^{+}$ TRBC1$^{+}$ | 2.8% | | HD with one clonal LGL population vs. HD |
| #4 | Monoclonal | PCR (whole PB) | Polytypic (0.45) | CD2$^{+}$ CD3$^{+}$ CD4$^{-}$ CD5$^{+}$ CD7$^{het}$ CD8$^{+}$ CD28$^{het}$ CD38$^{++}$ CD45RA$^{-}$ CD45RO$^{+}$ CD56$^{-}$ CD57$^{-}$ | 34% | Acute EBV infection | Reactive T-cell lymphocytosis |
| #5 | Monoclonal / Polyclonal | PCR (whole PB) / TCRVβ by FCM | Polytypic (0.37) | CD2$^{lo}$ CD3$^{+}$ CD4$^{-}$ CD5$^{-/lo}$ CD7$^{-/lo}$ CD8$^{lo}$ CD11c$^{+}$ CD28$^{-}$ CD45RA$^{+}$ CD45RO$^{-}$ CD56$^{-}$ CD57$^{-}$ | 62% | Neutropenia + lymphocytosis | Unclassifiable |

Abbreviations (alphabetical order): cy, cytoplasmic; FCM, flow cytometry; Gra, granzyme B; HD, healthy donor; het, heterogeneous expression; LGL, large granular lymphocytes; lo, low expression; *n*., number; PCTCL, primary cutaneous T-cell lymphomas; PCR, polymerase chain region; Perf, perforin; PB, peripheral blood; SS, Sézary syndrome; T-LGLL, T-cell large granular lymphocyte leukemia.

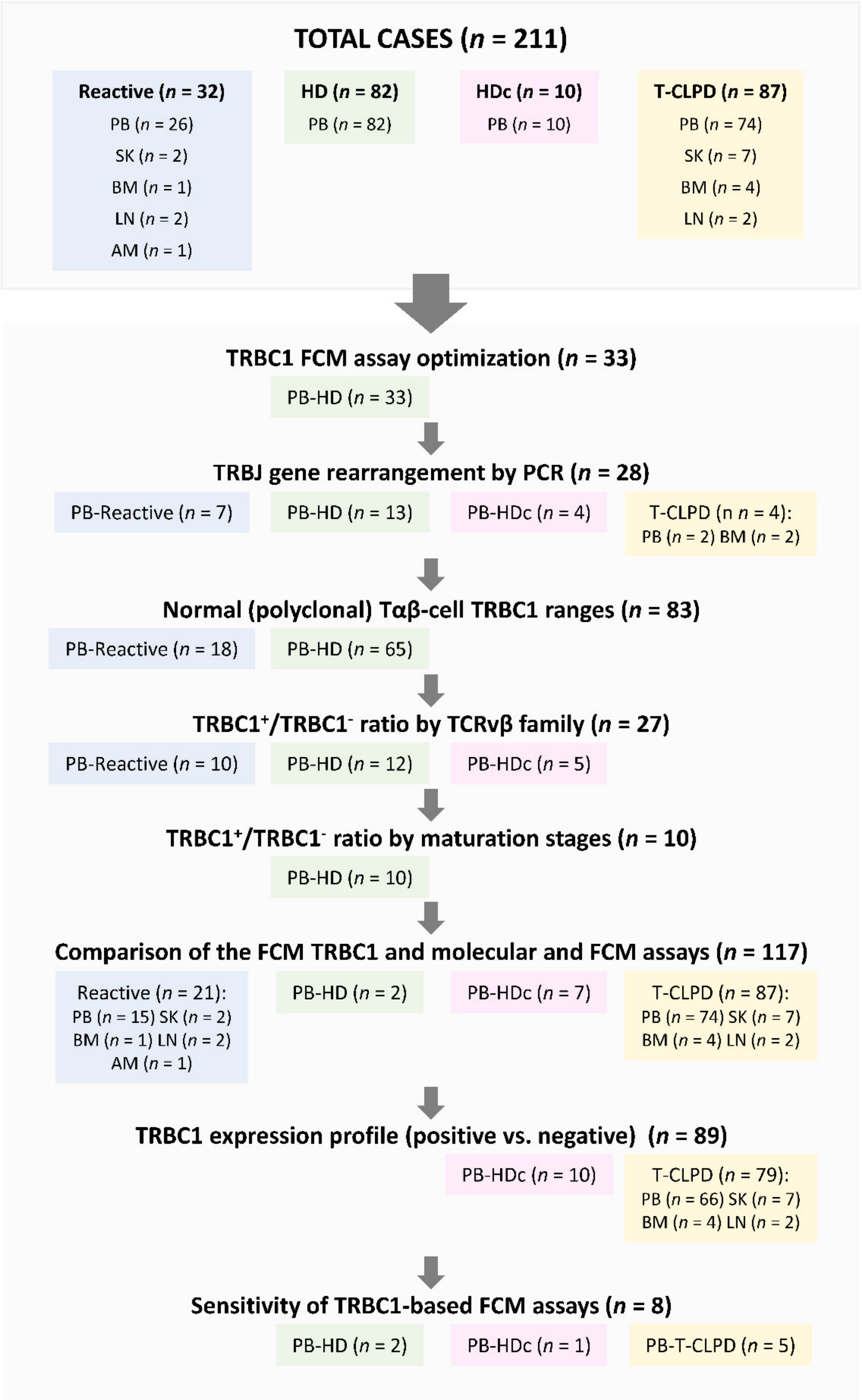


**Figure S1.** Flowchart illustrating the distribution of samples (*n* = 211, including 192 PB, 9 SK, 5 BM, 4 LN and 1 AM) and the study groups corresponding to the different sets of experiments performed. Abbreviations (alphabetical order): AM, abdominal mass; BM, bone marrow; FCM, flow cytometry; HDc, healthy donor with a small Tαβ-cell clone in blood; LN, lymph node; *n*, number of samples; PB, peripheral blood; SK, skin; T-CLPD, T-cell chronic lymphoproliferative disorders.

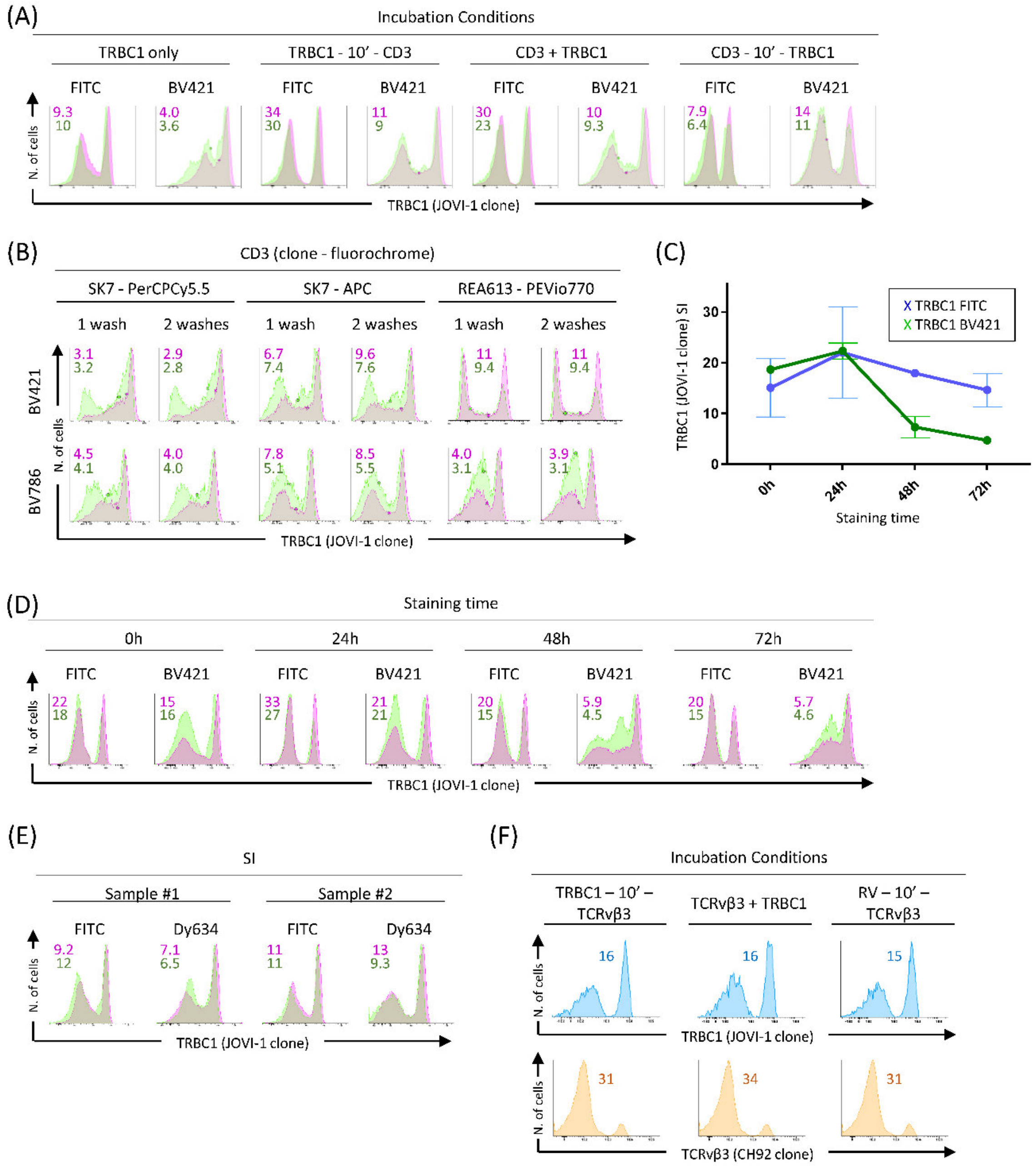


**Figure S2.** Pattern of expression of TRBC1 on blood T-cells using different staining conditions**.** (**A, B, D, E**) Illustrating histograms of the pattern of expression of TRBC1 for TαβCD4+ (depicted in pink) and TαβCD8+ (painted in green) cells; numbers inside the plots represent the corresponding TRBC1 stain index (SI). **(A)** Impact of CD3 staining on the TRBC1 expression profile: a representative blood sample stained under the four different incubation conditions tested for CD3-APC (clone SK7) and either a FITC- or BV421-fluorochrome conjugated TRBC1 reagent. **(B)** Impact of the number of washing steps (1 vs. 2) on the TRBC1 expression profile: a representative blood sample processed with either 1 or 2 washes after FACSLysing solution is shown. **(C, D)** Comparison of different time of incubation with the TRBC1 antibody reagent (at 0, 24, 48, 72 h after blood collection) on the pattern of expression of TRBC1: **(C)** TRBC1 SI on Tαβ-cells from two paired blood samples stained for TRBC1 using both FITC- and BV421-conjugated reagents; **(D)** Illustrating histogram plots from one representative blood sample for different staining times. **(E)** Comparison between FITC and Dy634 conjugated TRBC1 reagents: illustrating histograms from one representative blood sample. **(F)** Comparison of different staining conditions for the TRBC1 and TCRVβ antibody reagents: illustrating histogram plots of one representative blood sample stained using different staining conditions for the TCRVβ3+ vs. TRBC1-Dy634 staining assays described in Figure 1 (Panels F and G); numbers inside the plots represent the corresponding TRBC1 SI of TCRVβ3+ Tαβ-cells (painted in light blue) or total Tαβ-cells (depicted in orange). Stain index (SI) was calculated as ($MFI_{PP}$ - $MFI_{NP}$) / 2 x $rSD_{NP}$ where MFI represents median fluorescence intensity values (arbitrary units scaled from 0 to 262,144) and rSD means robust standard deviation, while PP and NP are used as abbreviations for the TRBC1 positive and the TRBC1 negative Tαβ populations, respectively.

Abbreviations (alphabetical order): APC, allophycocyanin; BV, brilliant violet; Cy, cyanin; Dy, dyomics; FITC, fluorescein-5-isothiocyanate; *n*., number; PE, phycoerythrin; PerCP, peridinin-chlorophyll-protein; SI, stain index.

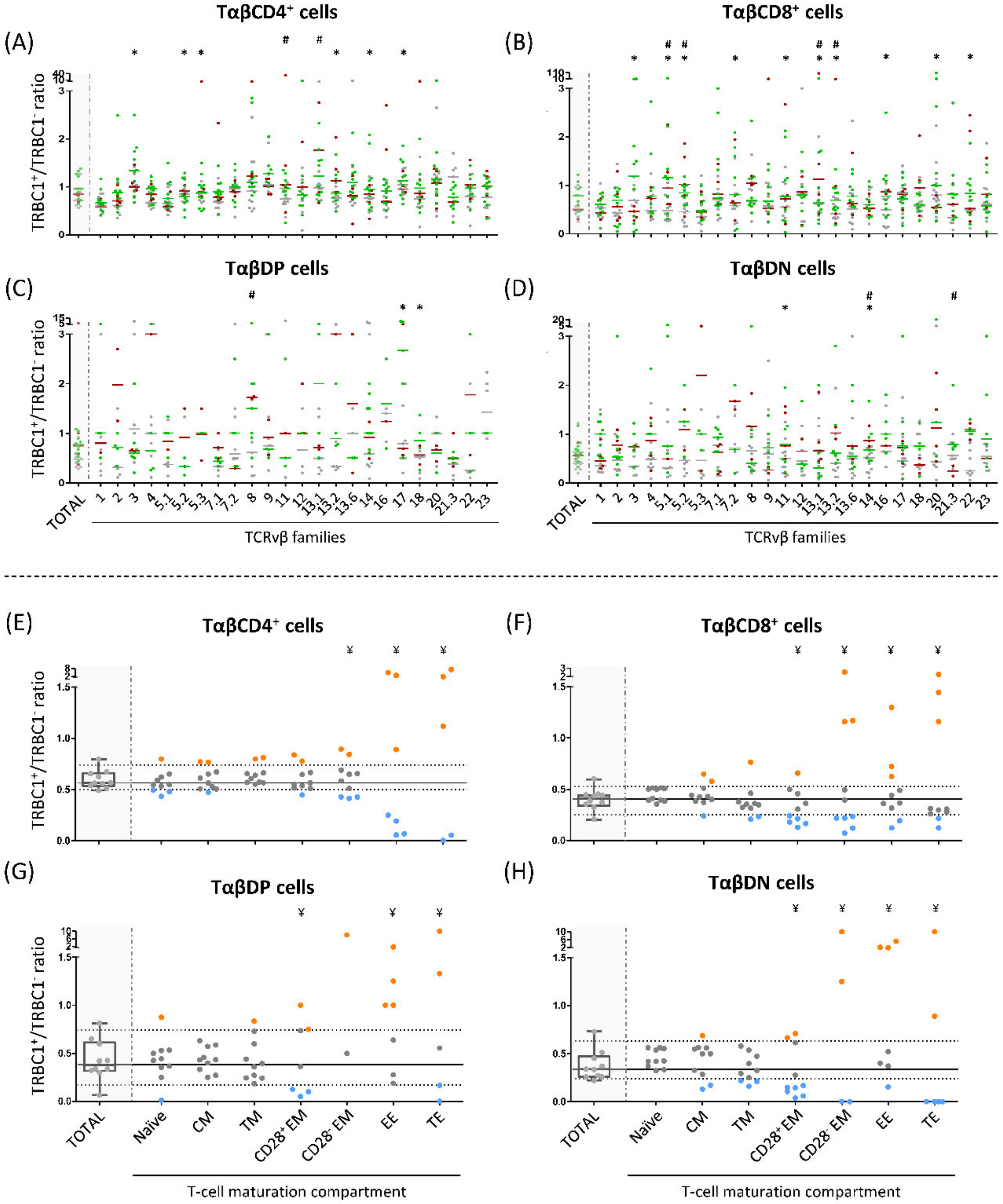


**Figure S3.** TRBC1+/TRBC1- ratio of normal Tαβ-cell subsets defined within the major TCD4, TCD8, TDP and TDN cell populations based on the pattern of expression of specific TCRVβ family members and their maturation stage. TRBC1+/TRBC1- ratio of the major TαβCD4+ (**A**), TαβCD8+ (**B**), TαβDP (**C**) and TαβDN (**D**) cell subsets within each of their subsets expressing different TCRVβ families in 12 HD (gray dots), 10 patients with reactive lymphocytosis (green dots) and 5 otherwise healthy individuals showing clonal expansions of Tαβ-cells in blood (HDc, red plots). In these latter subjects, the TCRVβ clonal population was removed from analysis. Colored horizontal lines are median values of the corresponding group of subjects. *p*-value ≤0.05 for * reactive lymphocytosis vs. HD and for # otherwise-HD with a T-cell clone vs. HD. TRBC1+/TRBC1- ratios for TαβCD4+ cells expressing different TCRVβ families ranged from 0.67 to 1.3 for blood samples from patients with reactive lymphocytosis and from 0.58 to 1.8 for HDc, with a significantly different distribution (vs. HD samples) for 6/24 (25%) and 2/24 (8%) TCRVβ families among cases with reactive lymphocytosis and HDc, respectively (Figure S3). For the TαβCD8+ cell subsets expressing different TCRVβ families, the TRBC1+/TRBC1- ratios ranged between 0.48 and 1.2 in blood samples from patients with reactive lymphocytosis and between 0.43 and 1.1

among HDc, a total of 10/24 (42%) and 4/24 (17%) TCRVβ families showing altered values (vs. normal PB) in reactive lymphocytosis and HDc patient samples, respectively (Figure S3). For other less represented subsets of Tαβ-lymphocytes, such as TαβDP and TαβDN cells, the TRBC1+/TRBC1- ratios ranged from 0.33 to 2.7 and from 0.31 to 1.3 for reactive lymphocytosis and from 0.29 to 3.0 and from 0.25 to 2.2 for HDc, respectively (Figure S3). In panels **E** to **H** the TRBC1+/TRBC1- ratio observed for normal TαβCD4+, TαβCD8+, TαβDP and TαβDN cells from 10 HD, distributed into different maturation-associated T-cell compartments is shown, respectively. ¥*p*-value ≤0.05 vs. the total populations. In all panels, dots correspond to results from individual experiments, while notched boxes represent 25th and 75th percentile values, lines inside the box correspond to median values (50th percentile) and whiskers represent minimum and maximum values. The continuous horizontal and dotted lines that cover the entire graph correspond to median values (50th percentile), and both the 5th and 95th percentile (P5 and P95), respectively. Cases above P95 are colored orange, while cases below P5 are shown in blue. Abbreviations (alphabetical order): CM, central memory; DN, double negative (TαβCD4-CD8-/lo) Tαβ-cells; DP, double positive (TαβCD4+CD8+) Tαβ-cells; EE, early effector; EM, effector memory; HD, healthy donor; HDc, healthy donor with a small Tαβ-cell clone in blood; TE, terminal effector; TM, transitional memory; Treg, regulatory T-cells.

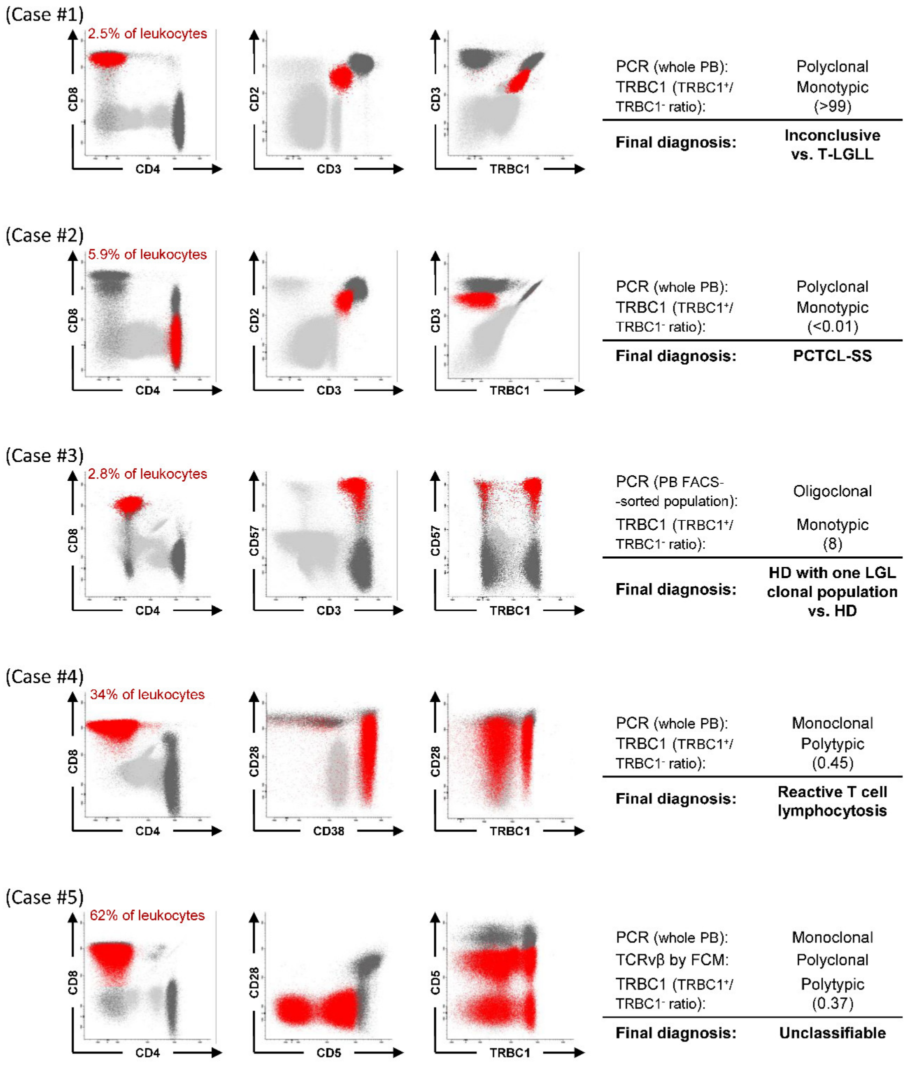


Distribution of TCRvβ regions by FCM

| TCRvβ | 1 | 2 | 3.1 | 4 | 5.1 | 5.2 | 5.3 | 7.1 | 7.2 | 8 | 9 | 11 | 12 | 13.1 | 13.2 | 13.6 | 14 | 16 | 17 | 18 | 20 | 21.3 | 22 | 23 |
|---|---|---|---|---|---|---|---|---|---|---|---|---|---|---|---|---|---|---|---|---|---|---|---|---|
| % of aberrant/ pathological cells | 2.1 | 0.44 | 4.0 | 0.99 | 4.5 | 0.40 | 0.45 | 1.3 | 0.89 | 3.7 | 14 | 0.61 | 1.7 | 1.2 | 1.5 | 1.1 | 4.6 | 9.9 | 2.2 | 0.39 | 2.3 | 1.9 | 1.4 | 0.21 |
| Σ TCRvβ = 62% | | | | | | | | | | | | | | | | | | | | | | | | |

**Figure S4**. Illustrative dot-plot diagrams, clonality data and final diagnosis of cases showing discrepant results between the pattern of expression of TRBC1 by FCM and the T-cell clonality status by conventional TCRVβ-FCM and/or molecular reference techniques (*n* = 5/117; 4.3%). Discrepant patients with an abnormal/suspicious population on phenotypic grounds (red dots) among residual normal T-cells (dark gray dots) and leukocytes (light gray dots) are numered #1 through #5 (as in Table S6). Abbreviations (alphabetical order): FCM, flow cytometry; HD, healthy donor; LGL, large

granular lymphocytes; PCTCL, primary cutaneous peripheral T-cell lymphoma; PB, peripheral blood; SS, Sezary syndrome; T-LGLL, T-cell large granular lymphocyte leukemia.

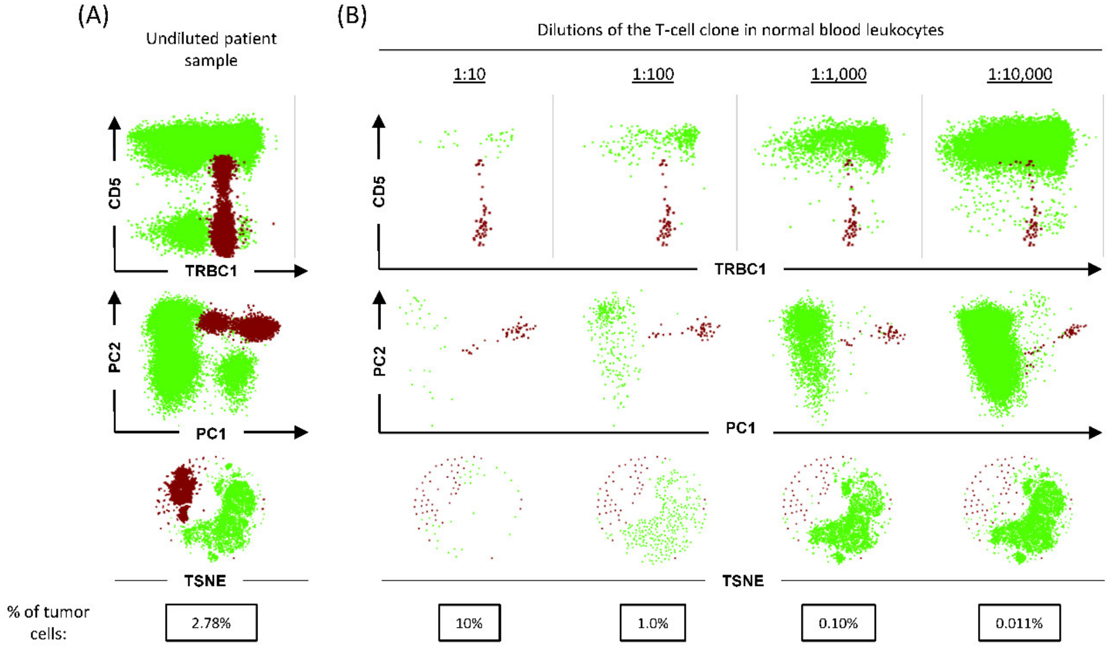


**Figure S5.** Illustrative dot-plot diagrams of a representative dilutional experiment of clonal Tαβ-cells from a T-LGLL patient in normal blood. (**A**) Undiluted blood sample from a TCD8$^+$ LGLL patient carrying a monoclonal T-cell expansion (dark red dots) among residual normal TCD8$^+$ cells (green dots). (**B**) The monoclonal TαβCD8$^+$ TCRVβ22$^+$ cell population (dark red dots) extracted from this TCD8$^+$ LGLL patient, was diluted in silico with normal blood TαβCD8$^+$ cells from a healthy donor (green dots) at the 1:10, 1:100, 1:1000 and 1:10,000 tumor cell/normal cell ratios. Bivariate CD5 vs. TRBC1 dot-plot diagrams are shown in the upper part of panels A and B, while the plots in the middle represent multidimensional (PC1 vs. PC2) representations of the phenotypic differences observed between the clonal and normal CD8$^+$ T-cells. Plots in the bottom panels correspond to TSNE diagrams of the same cells. Framed numbers represent the percentage of clonal TαβCD8$^+$ cells from all leukocytes identified in each experiment. Abbreviations (alphabetical order): HD, healthy donor; PC, principal component; T-LGLL, T-cell large granular lymphocyte leukemia; TSNE, t-distributed stochastic neighbor embedding.
